# Hyperuricemia was associated with metabolic response against brain injury instead of metabolism syndrome in Tibet: a cross-sectional analysis

**DOI:** 10.3389/fendo.2026.1776220

**Published:** 2026-04-23

**Authors:** Xue-Wen Ren, Qiu-Ying Zhang, Qing Chang, Ji-Wei Hao, Yu-Xin Feng, Qing-Lei Zhu, Qing-Hong Zhang

**Affiliations:** 1 Department of Emergency Medicine, First Medical Center of Chinese People’s Liberation Army General Hospital, Beijing, China; 2 Outpatient Department of the 54th Cadres’ Recuperation Center, Beijing Military Region, Beijing, China; 3 Laboratory Medicine, First Medical Center of Chinese People’s Liberation Army General Hospital, Beijing, China; 4 Department of Medical Innovation Study, State Key Laboratory of Trauma and Chemical Poisoning, Chinese People’s Liberation Army General Hospital, Beijing, China; 5 Department of Otolaryngology, First Medical Center of Chinese People’s Liberation Army General Hospital, Beijing, China; 6 Department of Cardiology, First Medical Center of Chinese People’s Liberation Army General Hospital, Beijing, China

**Keywords:** brain injury, glucagon-like peptide-1, hyperuricemia, insulin resistance, metabolic syndrome, Tibet (China), xanthine oxidoreductase

## Abstract

**Background:**

To determine if hyperuricemia be a metabolic response to prevent brain injury in high-altitude adaption in Tibet.

**Methods:**

This cross-sectional study enrolled 3 ethnics of males (18–35 yo) from 3 ascending altitudes (*n* = 1133). Metabolic and brain injury biomarkers were compared among the 3 ethnics, and between the normal (NUA) and high serum uric acid (HUA) groups.

**Results:**

Serum UA levels were simultaneously increased with altitudes in the 3 ethnics (all *P* < 0.0001), higher in the Hans and Minority than in the Tibetans (both: *P* < 0.0001). Exclusively in the Hans, xanthine oxidoreductase (XOR) activity was increased (*P* < 0.0001) whereas fractional excretion of UA was decreased (*P* < 0.0001) with altitudes, meanwhile, HOMA-IR (*P* < 0.0001) and HOMA-β (*P* = 0.017) were invariably reduced, and GLP-1(1-37a) (*P* < 0.0001) was consistently elevated with the altitudes. Beneficially, arteriosclerosis index was elevated with the altitudes in both the Tibetans and the Minorities rather than in the Hans. The level of pan-axonal marker, PGP 9.5, was elevated with altitudes only in the Hans (*P* = 0.007), by contrast, the astrocyte marker GFAP were elevated in both the Tibetans (*P* = 0.0003) and the Minorities (*P* = 0.038) but not in the Hans (*P* = 0.13). In comparison of HUA with NUA, while the Hans in shorter stay Lhasa-Yumai showed higher XOR, HOMA-IR, GLP-1(1-37a) and PGP 9.5 levels, PGP9.5 was lower in the Hans in longer stay Lhasa-Tolun; yet both neural biomarkers were elevated in the Tibetans of Lhasa, and tended to decline in Naqu.

**Conclusion:**

Hyperuricemia may follow distinctive neuroendocrine mechanism among the 3 ethnics to protect the brain from oxidative stress in Tibet.

## Graphical Abstract

**Figure:**
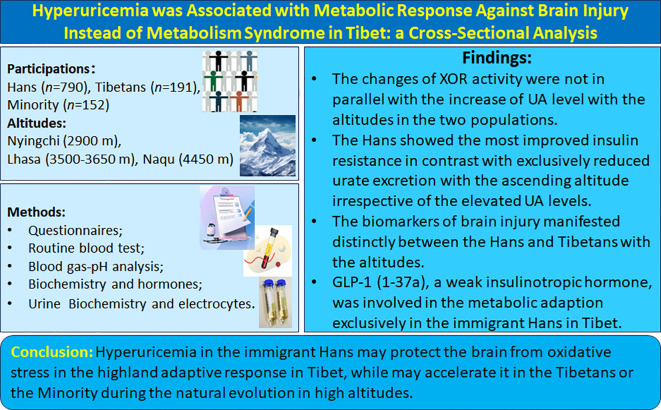

## 1 Introduction

Hyperuricemia prevails in Tibet which increases with the altitude in both the native Tibetans (1, 2) and the inland immigrants (1, 3), who even developed hyperuricemia within 1 month from low altitude to high altitude (4). Although the biochemistry profiles associated with hyperuricemia were distinctive between the Hans and the Tibetans (1), most people with hyperuricemia had not received routine anti-hyperuricemia therapy before the onset of gout because of the unknown etiology.

Hyperuricemia has always been recognized as part of metabolic syndrome (MS) that persistent metabolic abnormalities were risk factors for hyperuricemia (5, 6). In humans, fasting insulin concentrations were positively associated with serum uric acid (SUA) levels (7), while reducing the glycemic index over 5-weeks’ diet could lower SUA level (8). In animals, UA secretion from whole adipose tissue was increased in obese mice (9). On the other way, some evidences indicated that hyperuricemia may also lead to insulin resistance (IR). High UA (HUA)-induced glucose intolerance in hepatic macrophages contributed to IR with impaired insulin signaling pathway in mice (10). In kidney transplant recipients, HUA was strongly associated with increased risk of post-transplantation diabetes mellitus (11). More directly, the inhibitor of xanthine oxidoreductase (XOR), an enzyme catalyzes the oxidation of hypoxanthine to xanthine and then xanthine to UA in the purine degradation pathway (12), provided protective effects on insulin secretory capacity in patients with type 2 diabetes (13). Although the two processes are intimately intertwined, there has been no direct causal link between IR and hyperuricemia. Recently, evidence even emerged against causality in the association of diabetes with hyperuricemia. SUA did not track with the changes in insulin sensitivity, β-cell function, or glycemia in women with recent gestational diabetes (14). Elevated serum XOR activity, rather than SUA concentration, was associated with an increased risk of type 2 diabetes in diabetes-free adults (15). Nonetheless, the prevailing hypothesis of the hyperuricemia associated with IR stemmed from the evidence in humans with MS, which may not be optimal to be applied to the inland immigrants who were generally healthy before recruited to Tibet (4).

UA is a strong endogenous antioxidant that neutralizes the toxicity of reactive species on the neurovascular unit generated in acute brain ischemia of rats (16). During an acute ischemic stroke, the rapid reduction of UA levels at admission (17) and the consequent lower ratio of SUA to serum creatinine (18) correlated closely with stroke severity and a worse outcome in patients. On the contrary, the longitudinal study during a median follow-up of 9.02 years yielded a high risk of cumulative SUA exposure for incident stroke risk (19). A U-shaped relationship between SUA levels and all-cause mortality in patients with hypertension indicated moderate SUA levels may exert protective effect on the survival (20). Although high UA diet triggers the neuroinflammatory pathway in the hippocampus of the humans and the rats (21), exogenous UA supplementation could counteract the progression of redox-mediated ischemic brain damage in preclinical study (22). Even in poor-grade subarachnoid hemorrhage patients, pentose phosphate pathway (PPP) was a crucial target for sedatives to divergently modulate brain metabolism that associated with favorable clinical outcome (23). The above divergent data suggested the distinct etiology of SUA between acute stress response and chronic metabolic adaption in brain injury.

The potential brain injury in the extreme altitudes confers the lowlanders greater sensitivity to psychoneurological symptoms, including chronic mountain sickness (24), migraine headache (25), ataxic gait (26), and executive function and memory deficits (27). Accumulating evidence suggested that metabolic adaptations underpin human evolution to life at high altitude (28, 29), with cerebral hypometabolism that coordinating the suppression of energy demand (30, 31). In hypoxia-tolerant Quechua, the natives of high Andes, a relatively large fraction of the glucose taken up by the brain was released as lactate, meanwhile, lower glucose metabolic rates of the brain were found in Quechuas than in lowlanders (30). Distinct from the hypoxia-tolerant evolution, acute hypoxia exposure in healthy young males of lowland could elevate the cerebral perfusion and metabolic rate, which may represent an increase in neuronal activity (32).

Notwithstanding these uncertainties, we supposed that the high prevalence of hyperuricemia, especially the rapid increase of SUA in the immigrants may represent an early metabolic response to prevent brain injury when ascending to high altitude rather than a consequence of MS by themselves. To this end, we enrolled three cohorts of healthy young males including native Tibetans, immigrant Hans and Minorities in three altitudes of Chinese Tibetan region. We compared the hypoxia severity, metabolic changes, pancreas secreting and kidney filtrating capacity, and finally brain injury biomarkers among them, and between the groups with normal UA (NUA) and HUA in respective altitude. We aim to explore the pathophysiological significance of SUA in the high-altitude assimilation to reduce the worry about the high prevalence of hyperuricemia in highland immigrants, in the meantime, to provide an alternative strategy to reduce its adverse effect in Tibet.

## 2 Materials and methods

### 2.1 Study design, ethical proof and the enrollment of the populations

This study was based on annual health check-up of local university students and servicemen. Male adults (18–35 years old) from the Hans, Tibetans and Minority were recruited from three different geographical cities with ascending altitudes in Chinese Tibetan region in December 2023. Since hyperuricemia prevails more severely in the males than in the females (33), we only recruited the males for the priority in the study, nonetheless, the findings in males are expected to be relevant for the females. The young populations were chosen because they are generally healthy that may exclude the confounding factors. Secondly, their physiological condition could truly reflect the adaptive changes to highland Tibet. Thirdly, the Minority population with natively higher attitude of southwest China was supposed to be more capable to adapt to the extreme environment than the inland Hans.

We applied for the ethical approval from our hospital ethics board with proof number (S2024-212-01). Our team first flied to Lhasa and enrolled the local participants for annual health check-up, then moved to Nyingchi and Naqu. The participants were fully aware of the study and were asked to be fasted overnight and collect the urine in the early morning. They signed the informed consent on hospital admission. Health questionnaires, clinical measurements, oral glucose tolerance test (OGTT), and laboratory examinations were carried out following standardized protocols. The questionnaires on immigrant time, eating behavior, and health status were completed under the instruction of chief doctor (XWR).

### 2.2 Procedures and sample collection

After an overnight fast, urine samples were collected into a 15 mL Eppendorf microfuge in the early morning and kept at room temperature before transferred to clinical laboratory. Anthropometric parameters and blood pressure were measured by standard methods (Omron HEM-770A) around 0800 h. Then 10 mL venous blood was drained from basilic vein in the forearm between 0800 h and 0900 h into one EDTA-K2 tube (purple) for routine blood test, two heparinized tubes (green) for pancreatic β cells secretion and gas analysis respectively, and one tube without anticoagulant (red) for biochemistry analysis (BD Vacutainer^®^, BD Pharmingen, USA). The rest blood was collected in one heparinized tube (green) and another tube without anticoagulant (red) for plasma and serum separations, respectively. The two tubes were stored in -20 °C and transferred in dry ice to Beijing and stored at -80 °C for XOR and glucagon-like peptide-1 (GLP-1) analysis.

Immediately after the blood collection, each participant underwent a 2h OGTT by taking 100 g steamed bun meal. The blood samples collected at 0 h in EDTA-K2 tube (purple) and those at 2 h of OGTT in heparinized tube (green) were centrifuged at 4,000 rpm×10 min within 4 h of collection, the supernatant plasma were aspirated and stored at -20 °C.

From EDTA-K_2_ tube, routine blood test was carried out immediately by a hematology analyzer (BF6900 CRP, Di-Rui INDUSTRIAL CO., LTD., Changchun, Jilin, China). From heparinized tubes, the venous blood gases were determined using a blood gas-pH analyzer (Ciba Corning 248 blood gas-pH analyzer, Medford, MA, USA), and the plasma was separated and stored at -20 °C for pancreatic β cells secretion analysis (Norson medical laboratory, Chengdu, Sichuan, China). From tubes without anticoagulant (red), the serum was separated for biochemistry analysis (Norson medical laboratory).

### 2.3 Blood and urine analysis

All laboratory analyses were conducted in a “blinded” manner. Fasting C-peptide, HbA1c, fasted and 2h OGTT glucose and insulin levels were immediately measured. Within 3 days of collection, the samples were subjected to automatic analysis of biochemistry profile by standard clinical chemistry laboratory methods (Cobas 8000, Roche, USA) (Norson Medical Laboratory, Chengdu, Sichuan, China) as previously reported (1). Venous blood lactate and electrolytes levels were detected by gas analysis (HC9885, Shenzhen Xi-Lai-Heng Medical Electronics Co., Ltd, Guangdong, China). Meanwhile, urine UA, creatinine, and electrolytes were also analyzed (Norson medical laboratory).

Plasma levels of serum XOR including two interconvertible forms of xanthine dehydrogenase and xanthine oxidase (Amplex^®^ Red Xanthine/Xanthine Oxidase Assay Kit, Cat.A22182, Molecular Probes, Invitrogen Detection Technologies, Eugene, OR, USA), human GLP-1 (1-37a) (Cat.EH221RB, Invitrogen, USA), and active human GLP-1 (7–36) (ab184857, Abcam, USA) were determined on an ELISA reader (ELx808, Biotek ELx808, USA) after incubated in a microplate oscillator (L-MPI-S-B, Labgic, Hefei, China). Serum protein gene product 9.5 (PGP 9.5) and glial fibrillary acidic protein (GFAP) levels were detected by chemiluminescence with commercial kits (Sophonix Co., Ltd, Beijing, China).

### 2.4 Assays and calculations

Pancreatic β-cell function was estimated by homoeostasis model assessment of β-cell function (HOMA-β) (34) calculated as 20× (fasting insulin [μU/mL])× (fasting glucose -3.5 [mmol/L]) (35). Insulin sensitivity was measured by the homoeostasis model assessment of IR index (HOMA-IR) as calculated as (fasting glucose[mmol/L])×(fasting insulin [μU/mL])/22.5 (36). The fractional excretion of uric acid (FEUA) was calculated as FEUA = (24h Uua × Scr)/(Sua × 24h Ucr) × 100 (37, 38). Uua and Sua represent urinary and serum UA, while Ucr and Scr is urinary and serum creatinine.

### 2.5 Statistical analysis

Hyperuricemia in males was defined as a fasting serum UA ≥ 420 μmol/L (39). GraphPad Prism 10.0 (GraphPad Software, USA) was used for data analysis. The normality of the distribution of the variables was assessed with the Shapiro-Wilk test. The mean and standard deviation (SD) were used to describe variables that met the normal distribution, and the median and interquartile (IQR) distance were used to describe variables that did not meet the normal distribution. We summarized baseline characteristics of participants as means (SD) or medians (IQR) for continuous variables and numbers (proportions) for categorical variables. Outliers were identified if they were >1.5 IQR ranges below the first quartile or above the third quartile. Participants with ≥3 missing data were excluded.

Differences between subjects were analyzed using the Chi-squared test or Fisher’s exact test for categorical data. The Hans and the Tibetans were subjected to Multivariate linear regression analysis to estimate the associations between the major indices and UA level. One-way ANOVA was performed for each variable in single ethnical population among the three altitudes. The differences among the three ethnic populations from the 3 different altitudes and their interactions were determined by mixed-effects analysis. When the main effect was significant, Tukey’s multiple comparisons test was performed. An unpaired *t* test was used for intergroup comparisons between the NUA and HUA.

## 3 Result

### 3.1 Characteristics of the three cohorts of population from three ascending altitudes

**Figure 1:**
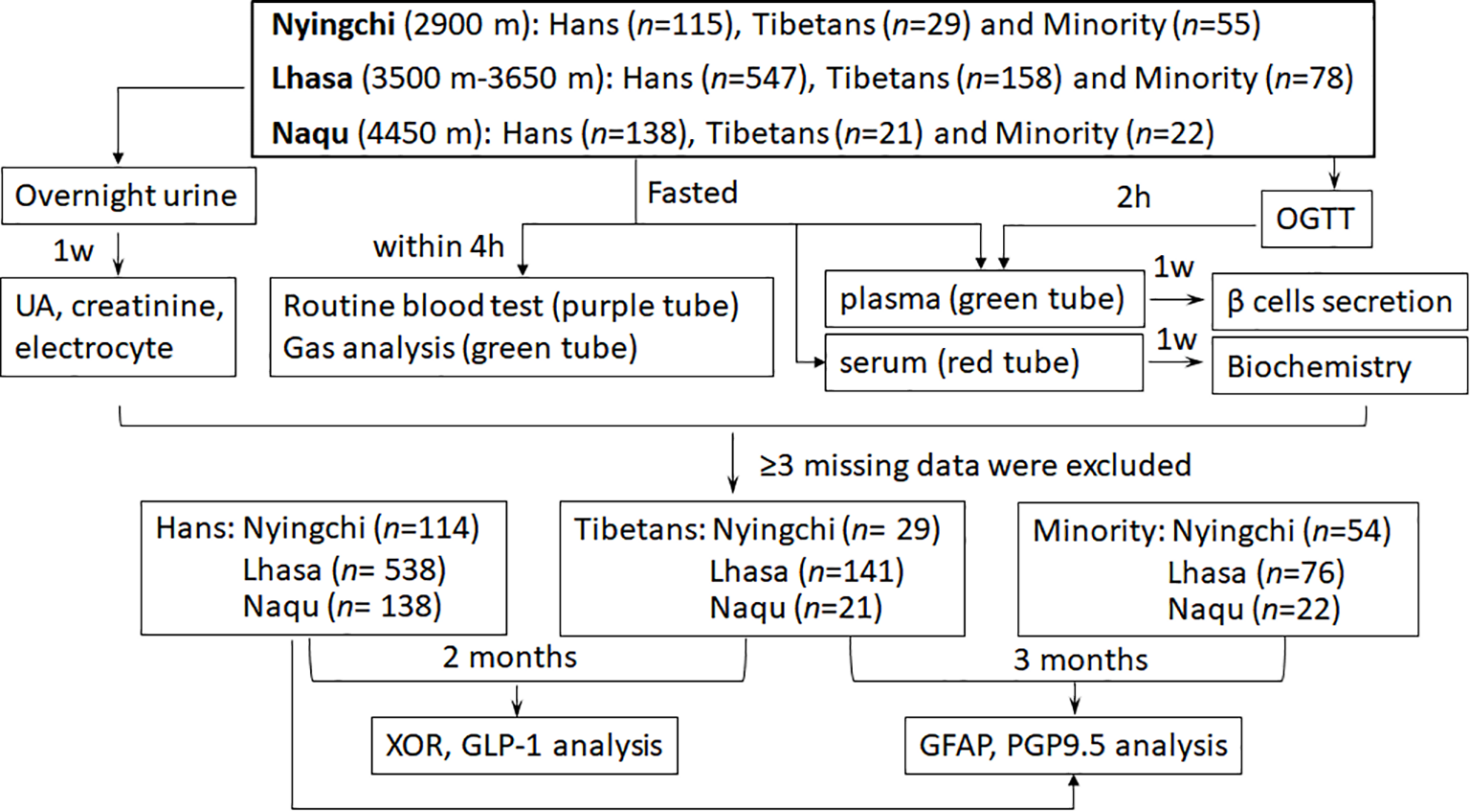
Overview of study cohorts, procedure, biochemistry and metabolic measurements. Footnote: XOR (xanthine oxidoreductase), UA (uric acid), GLP-1 (glucagon-like peptide 1), PGP 9.5 (Protein Gene Product 9.5), GFAP (glial fibrillary acidic protein), h (hour), w (week).

The survey was based on samples drawn from three cohorts of young male populations covering three different cities in Chinese Tibet region with ascending altitudes of Nyingchi at 2900 m, Lhasa at 3500–3650 m, Naqu at 4450 m (**Figure 1**). The participations with ≥3 missing data were excluded, and finally 790 Hans, 191 native Tibetans and 152 ethnic Minorities were included. Since the Han population stayed in the two suburban counties of Lhasa within quite different time, so we separated the Hans into “Lhasa-Tolun” (3650 m) and “Lhasa-Yumai”(3500 m) subgroups in the following study.

The dwelling time of the Hans was similar among Nyingchi, Lhasa-Tolun and Naqu, but was shortest in Lhasa-Yumai [0.42 (0.42-0.75) years], e.g., 5 (5–9) months. The 4 cohorts of Hans from the three altitudes covered the similar ages at 23 (20–26) (Nyingchi), 24 (22–26) (Lhasa-Tolun), 23 (22–26) (Lhasa-Yumai) and 23 (22–26) (Naqu) years (*P* = 0.38). The incidence of hyperuricemia was 57.0% in the total Han population (450/790) with a median (IQR) age (years) of 23 (22–26) and was increased significantly with the altitude, 41.2% (47/114) in Nyingchi, 48.9% (112/229) in Lhasa-Tolun, 58.3% (180/309) in Lhasa-Yumai, and 81.0% (111/137) in Naqu (*P* < 0.0001) (**Table 1**).

**Table 1:** Characteristic of the male populations from the three ethnics in three high-altitudes of Tibet, China.

Ethnicity	Location	Nyingchi	Lhasa-Tolun	Lhasa-Yumai	Naqu	Total	*P* value
	Altitude	2900 m	3630–3650 m	3500 m	4450 m		
	n	114	229	309	138	790	
	Age (y), median (IQR)	23 (22-26)	24 (22-26)	23 (22-26)	23 (22-26)	23 (22-26)	0.38
	Dwelling time (y),median (IQR)	3.29 (1.75-7.00)	3.00 (1.04-8.25)	0.42 (0.42-0.75)^a,b^	3.25 (1.25-6.25)^c^	1.75 (0.50-4.25)	<0.0001
	HUA incidence, n (%)	47 (41.2)	112 (48.9)	180 (58.3)	111(80.4)	450 (57.0)	<0.0001
	Height (cm)	171 (168-175)	173 (170-177)	175 (171-178)	172 (169-176)	173 (170-177)	<0.0001
	BMI (median, IQR)	22.1 (20.8-23.3)	22.2 (20.5-23.7)	22.0 (20.6-23.5)	22.1 (20.5-23.5)	22.1 (20.6-23.6)	0.96
	Heart Rate	62 (55-71)	73 (70-80)^a^	71 (64-80)^a^	76 (68-83)^a,c^	72 (65-80)	<0.0001
	Diet (1:vegetable,2:balanced,3:meat)						<0.001
	1	0	3	7	1	11	
Hans	2	110	182	262	127	681	
	3	4	43	40	10	97	
	Alcohol (1:never,2:seldom,3:often)						0.13
	1	32	86	106	41	265	
	2	80	139	203	96	518	
	3	2	4	0	1	7	
	Water intake (1<1000ml,2:1000-2000ml; 3≥2000ml)						0.06
	1	38	86	109	48	281	
	2	66	130	181	70	447	
	3	9	13	19	20	61	
	Smoke, n (%)	80 (70.8)	154 (67.3)	211 (68.5)	92 (66.7)	537 (68.0)	0.93
	Acute high-altitude response, n (%)	1 (0.9)	6 (2.6)	3 (1.0)	4 (2.9)	14 (1.8)	0.31
	Arthritis or other rheumatic diseases, n (%)	4 (3.5)	5 (2.2)	10 (3.2)	6 (4.4)	25 (3.2)	0.71
	Gout (n,%)	0 (0)	20 (8.7)	11 (3.6)	14 (10.1)	45 (5.7)	<0.001
	Gastropathy (n,%)	3 (2.6)	32 (14.0)	23 (7.4)	8 (5.8)	66 (8.4)	0.001
	n	29	141		21	191	
	Age (y), median (IQR)	23.0 (21.5-25.0)	22.0 (21.0-24.0)		24.0 (20.5-26.0)	22.0 (21.0-24.0)	0.11
	HUA incidence, n (%)	5 (17.2)	59 (41.8)		13 (61.9)	77 (40.3)	0.004
	Height (cm)	174 (170-176)	173 (170-176)		171 (165-180)	173 (170-176)	0.82
	BMI (median, IQR)	21.2 (20.6-23.2)	21.3 (20.0-22.5)		21.5 (20.6-24.7)	21.3 (20.1-22.6)	0.21
	Heart Rate	57 (53-62)	72 (65-79)a		73 (69-85)^a^	70 (63-78)	<0.0001
	Diet (1:vegetable,2:balanced,3:meat)						0.47
	1	0	9		0	9	
	2	23	106		16	145	
	3	6	26		5	37	
Tibetans	Alcohol (1:never,2:seldom,3:often)						
	1	9	67		7	83	
	2	20	74		14	108	
	3	0	0		0	0	
	Water intake (1<1000ml,2:1000-2000ml; 3≥2000m)						0.043
	1	6	55		2	63	
	2	18	68		14	100	
	3	5	18		5	28	
	Smoke, n (%)	21 (72.4)	89 (63.1)		14 (66.7)	124 (64.9)	0.62
	Acute high-altitude response, n (%)	0	0		0	0	
	Arthritis or other rheumatic diseases, n (%)	0	5 (3.55)		0	5 (2.6)	0.40
	Gout (n,%)	1 (3.5)	2 (1.4)		3 (14.3)	6 (3.1)	0.007
	Gastropathy (n,%)	0	7 (5.0)		4 (19.1)	11 (5.8)	0.013
	n	54	76		22	152	
	Age (y), median (IQR)	22 (21-24)	25.0 (22.3-26.8)a		25.5 (22.8-27.0)^a^	24 (22-26)	0.0002
	Dwelling time (y), median (IQR)	1.75 (1.17-3.25)	0.75 (0.42-2.42)a		5.75 (1.5-7.25)^b^	1.75 (0.75-4.25)	<0.0001
	HUA incidence, n (%)	24 (44.4)	46 (60.5)		17 (77.3)	87 (57.2)	0.030
	Height (cm)	170 (167-175)	172 (168-177)		170 (167-170)	170 (168-175)	0.032
	BMI (median, IQR)	21.7 (20.8-22.5)	22.3 (20.8-23.3)		21.1 (20.4-22.5)	21.9 (20.8-23.1)	0.19
	Heart Rate	61 (55-66)	72 (65-81)^a^		73 (66-84)^a^	69 (61-75)	< 0.0001
	Diet (1:vegetable,2:balanced,3:meat)						0.84
	1	1	1		0	2	
	2	50	67		20	137	
Minority	3	3	8		2	13	
	Alcohol (1:never,2:seldom,3:often)						0.84
	1	17	20		7	44	
	2	37	55		15	107	
	3	0	1		0	1	
	Water intake (1<1000 ml,2:1000–2000 ml; 3≥2000m)						0.16
	1	21	29		6	56	
	2	29	46		13	88	
	3	4	1		3	8	
	Smoke, n (%)	32 (59.3)	56 (73.7)		14 (63.6)	102 (67.1)	0.21
	Acute high-altitude response, n (%)	0	2 (2.6)		1 (4.6)	3 (2.0)	0.37
	Arthritis or other rheumatic diseases, n (%)	1 (1.9)	2 (2.6)		0	3 (2.0)	0.73
	Gout (n,%)	0	2 (2.63)		0	2 (1.32)	0.37
	Gastropathy (n,%)	4 (7.4)	8 (10.5)		0	12 (7.9)	0.27

IQR, interquartile range. ^a^*P* < 0.001 *vs.* Nyingchi, ^b^*P* < 0.001 *vs.* Lhasa-Tolun/Lhasa, ^c^*P* < 0.001 *vs.* Lhasa-Yumai. HUA, high uric acid, BMI, Body mass index.

The native Tibetans showed significantly increased incidence of hyperuricemia with the altitude, 17.2% (5/29) in Nyingchi, 41.8% (59/141) in Lhasa, and 61.9% (13/21) in Naqu (*P* < 0.0001) with similar ages among the three altitudes (*P* = 0.11).The total incidence of hyperuricemia was 40.3% (77/191) in the native Tibetans with a median (IQR) age of 22 (21–24) years. The Minorities shared comparable incidence of hyperuricemia as the Hans with total incidence of 57.2% (87/152), which was also increased with the ascending altitude (**Table 1**).

Every ethnic population had significantly increased heart rate with the ascending altitudes (all: *P* < 0.0001) (**Table 1**). The intake of diet, alcohol, water and smoke did not differ among the three altitudes significantly in any of the three ethnic populations. The incidences of arthritis or other rheumatic disease were similar among the three ethnic populations and among the different altitudes. However, the incidence of gout was higher in the Hans (5.7%) than in the Tibetans (3.1%) or the Minority (1.3%) and was highest in Naqu in both the Hans and the Tibetans (**Table 1**).

### 3.2 The increase of SUA was not in proportion with XOR levels in both the Hans and the Tibetans

**Figure 2:**
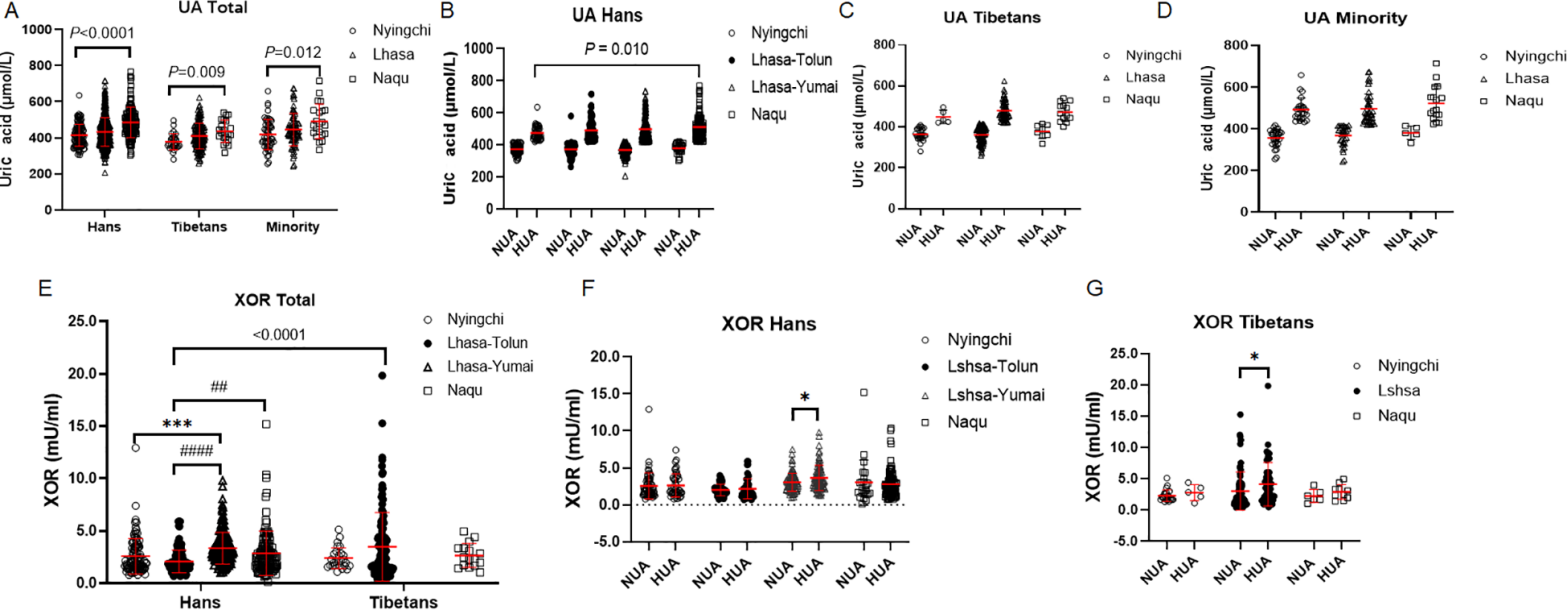
Comparison of serum uric acid (SUA) **(A–D)** and xanthine oxidoreductase (XOR) levels **(E–G)** among the Hans, Tibetans, and Minorities in the three ascending altitudes of Chinese Tibet region **(A,E)**, and between the respective subpopulations with normal uric acid (NUA) or high uric acid (HUA) levels in Tibet **(B–D, F–G)**. *P* values denote the difference among the different counties in the same ethnics or in the Hans with HUA as analyzed by one-way ANOVA. *P < 0.05, ***P < 0.001, *vs.* Nyingchi or NUA; ^##^*P* < 0.01, ^####^*P* < 0.0001 *vs.* Lhasa-Tolun by unpaired *t* test.

SUA in all the three ethnic populations were increased with the altitudes (Hans: *P* < 0.0001; Tibetans: *P* = 0.009; Minority: *P* = 0.012) with higher magnitudes in both the Hans and the Minority than in the Tibetans (both: *P* < 0.0001), but similar magnitudes between the Hans and Minority (*P* = 0.20) (**Figure 2A**). Furthermore, SUA was significantly increased with the altitude exclusively in the Hans of HUA population (*P* = 0.010) (**Figures 2B–D**).

Since the above manifestation of SUA was similar between the Hans and the Minority, we only compared XOR levels between the Hans and the Tibetans. Similar to XOR level as reported in male adults (Mean± SD: 4.00 ± 2.77 U/L) (40), XOR level was around 4 mU/ml in our study. It was increased with the altitudes in the Hans (*P* < 0.0001) but not in the Tibetans (*P* = 0.93), resulting in significant difference between the two ethnics (F (1, 671)=14.32, *P* = 0.0002), among the 3 altitudes (F(3, 671)=7.312, *P* < 0.0001) and interaction between the altitude and the two ethnics (*P* = 0.021) (**Figure 2E**). In detail, XOR levels were comparable between the Hans and the Tibetans in both the Nyingchi and Naqu. However, Tibetans in Lhasa had higher XOR level than the Hans in Lhasa-Tolun (*P* < 0.0001). Moreover, the changes of XOR with the altitude were not in proportion with the increase of SUA. Except that XOR was higher in HUA than in NUA in the Hans from Lhasa-Yumai (**Figure 2F**) and in the Tibetans from Lhasa, it did not differ between HUA and NUA groups in both ethnics from other altitudes (**Figures 2F, G**). Accordingly, SUA was associated with fasted glucose, HbA1C, and XOR exclusively in the Hans rather than in the Tibetans (**Supplementary Table 1**).

### 3.3 SUA was more associated with oxygen carrying capacity than with the hypoxia severity in all the three ethnic populations

**Figure 3:**
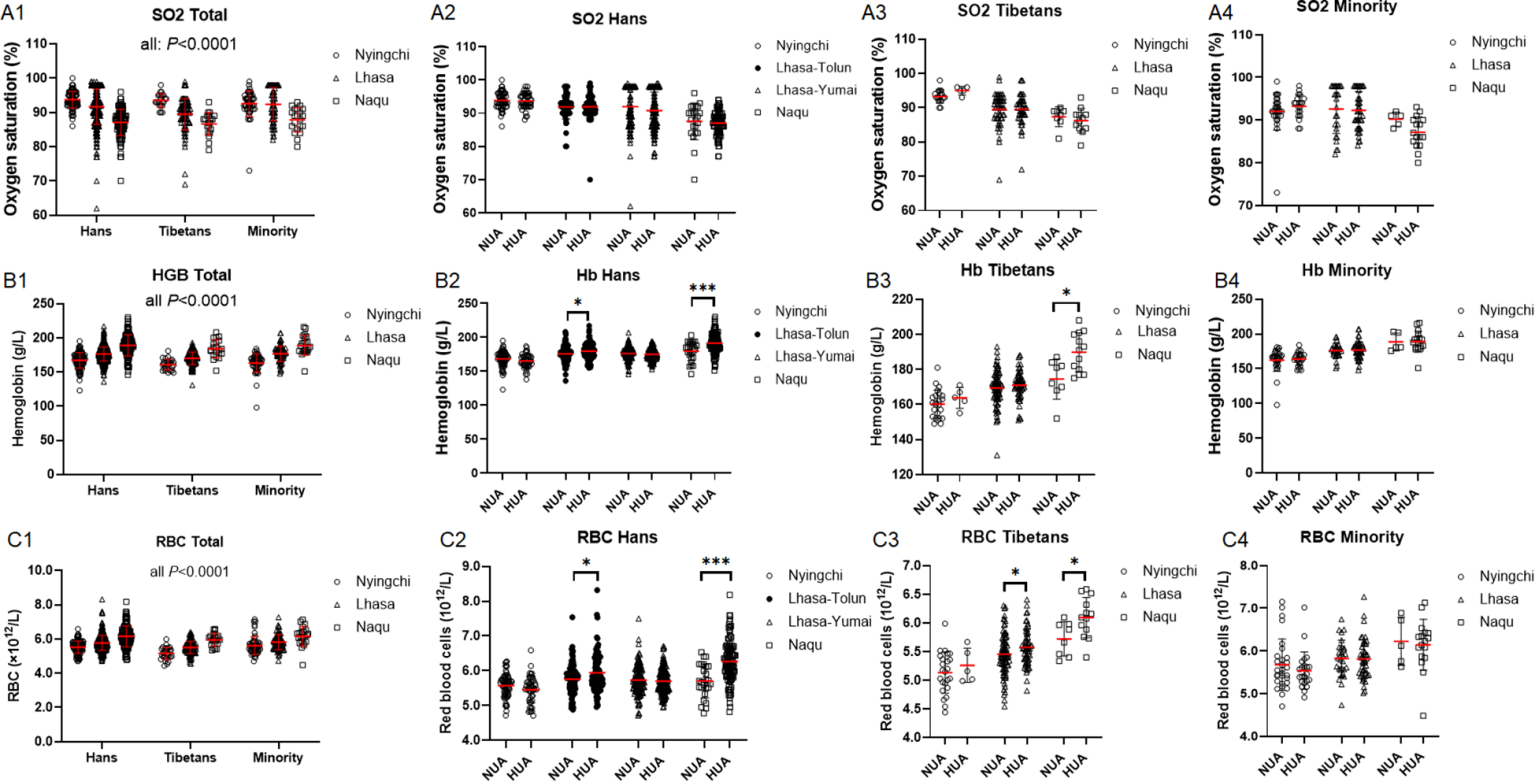
Comparison of oxygen saturation (SO_2_), **(A)**, hemoglobin (HGB) **(B)**and red blood cells (RBC) **(C)**among the Hans, Tibetans, and Minorities in three altitudes of Chinese Tibet, and between the respective subpopulations with normal uric acid (NUA) or high uric acid (HUA) levels in Tibet. *P* values denote the difference among the different counties in the same ethnicity as analyzed by one-way ANOVA. *P < 0.05, ***P < 0.001 *vs.* NUA by unpaired t test.

Oxygen saturations (SO_2_), the biomarker of hypoxia severity, were decreased with the altitudes in the 3 ethnic populations (all: *P* < 0.0001) at similar magnitude among the 3 ethnic populations (*P* = 0.18) (**Figure 3A1**). However, SO_2_ did not differ between the NUA and HUA groups in the 3 counties from the three ethnic cohorts (**Figures 3A2-4**). Inversely, both HGB and red blood cells (RBCs), which represent the oxygen carrying capacity, were increased with the altitudes in the 3 ethnic cohorts (all *P* < 0.0001) with higher magnitude in the Hans/Minorities than in the Tibetans (HGB: Hans vs. Tibetans: *P* < 0.0001, Hans *vs.* Minorities: *P* = 0.58, Tibetans *vs.* Minorities: *P* = 0.008; RBC: Hans/Minorities *vs.* Tibetans: *P* < 0.0001, Hans *vs.* Minorities: *P* = 0.58) (**Figures 3B1,C1**). Both the HGB and RBCs of Han population were higher in HUA than in NUA in Lhasa-Tolun and Naqu (**Figures 3B2, C2**). Similarly, the Tibetans also exhibited higher HGB and RBC levels in HUA than in NUA in both Lhasa and Naqu (**Figures 3B3, C3**). However, the two parameters were similar between NUA and HUA groups in the Minority (**Figures 3B4, C4**). However, peripheral mononuclear blood cells were not changed so much as RBCs (**Supplementary Figure 1**).

### 3.4 SUA was associated with glucose metabolism exclusively in the Hans

**Figure 4:**
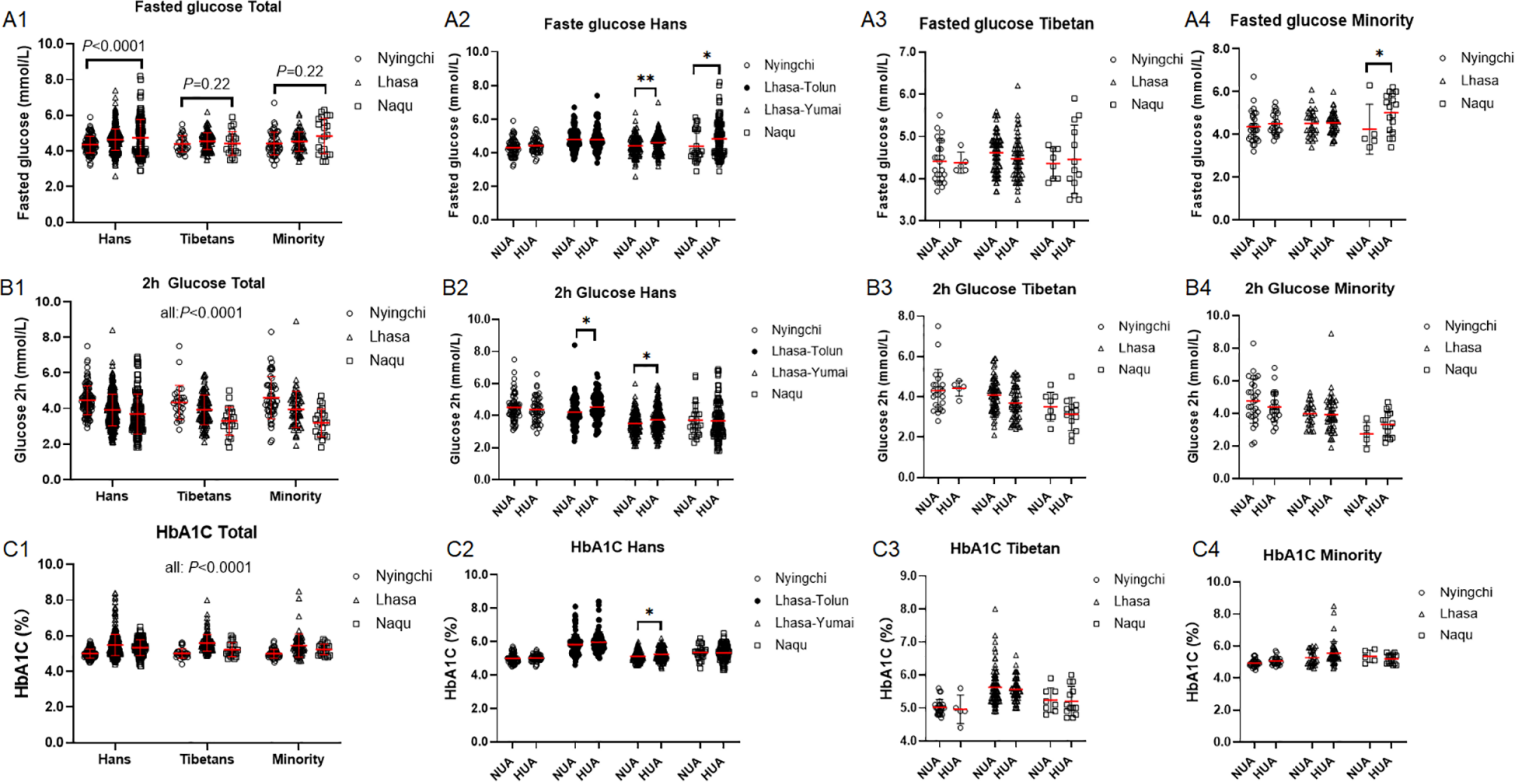
Comparison of glucose metabolism in oral glucose tolerance test among the Hans, Tibetans, and Minorities in the three altitudes of Tibetan region, **(A1–C1)**, and between the respective subpopulations with normal uric acid (NUA) or high uric acid (HUA) levels in Tibet region **(A2–4, B2–4, C2–4)**. *P* values denote the difference among the different counties in the same ethnicity as analyzed by one-way ANOVA. HbA1C: Glycosylated Hemoglobin, Type A1C. *P < 0.05, **P < 0.01 *vs.* NUA by unpaired t test.

The fasted glucose level was increased with the altitude in the Hans (*P* < 0.0001) rather than in both the Tibetans (*P* = 0.22) and the Minority (*P* = 0.22) (**Figure 4A1**). It was elevated in HUA than in NUA in the Hans of Lhasa-Yumai and Naqu (**Figure 4A2**), in the Minorities of Naqu (**Figure 4A4**), but not changed in the Tibetans at any altitude (**Figure 4A3**).

The OGTT 2h glucose levels were decreased with the altitudes in all the 3 ethnic cohorts (all: *P* < 0.0001) at similar magnitude between the Hans and the Tibetans (*P* = 0.19) (**Figure 4B1**). HUA group had higher OGTT 2h glucose level than NUA group exclusively in the Hans from the two counties of Lhasa (**Figures 4B2-4**), but similar OGTT 2h glucose levels in both the Tibetans and the Minorities.

HbA1C was increased with the altitude in the three ethnic populations (all: *P* < 0.0001) at similar magnitude between the Hans and the Tibetans (*P* = 0.57) (**Figure 4C1**). Once again, HUA group had higher HbA1C level than NUA group exclusively in the Hans of Lhasa-Yumai (**Figure 4C2-4**).

### 3.5 SUA was associated with higher insulin secretions depending on the ethnics and the altitudes

**Figure 5:**
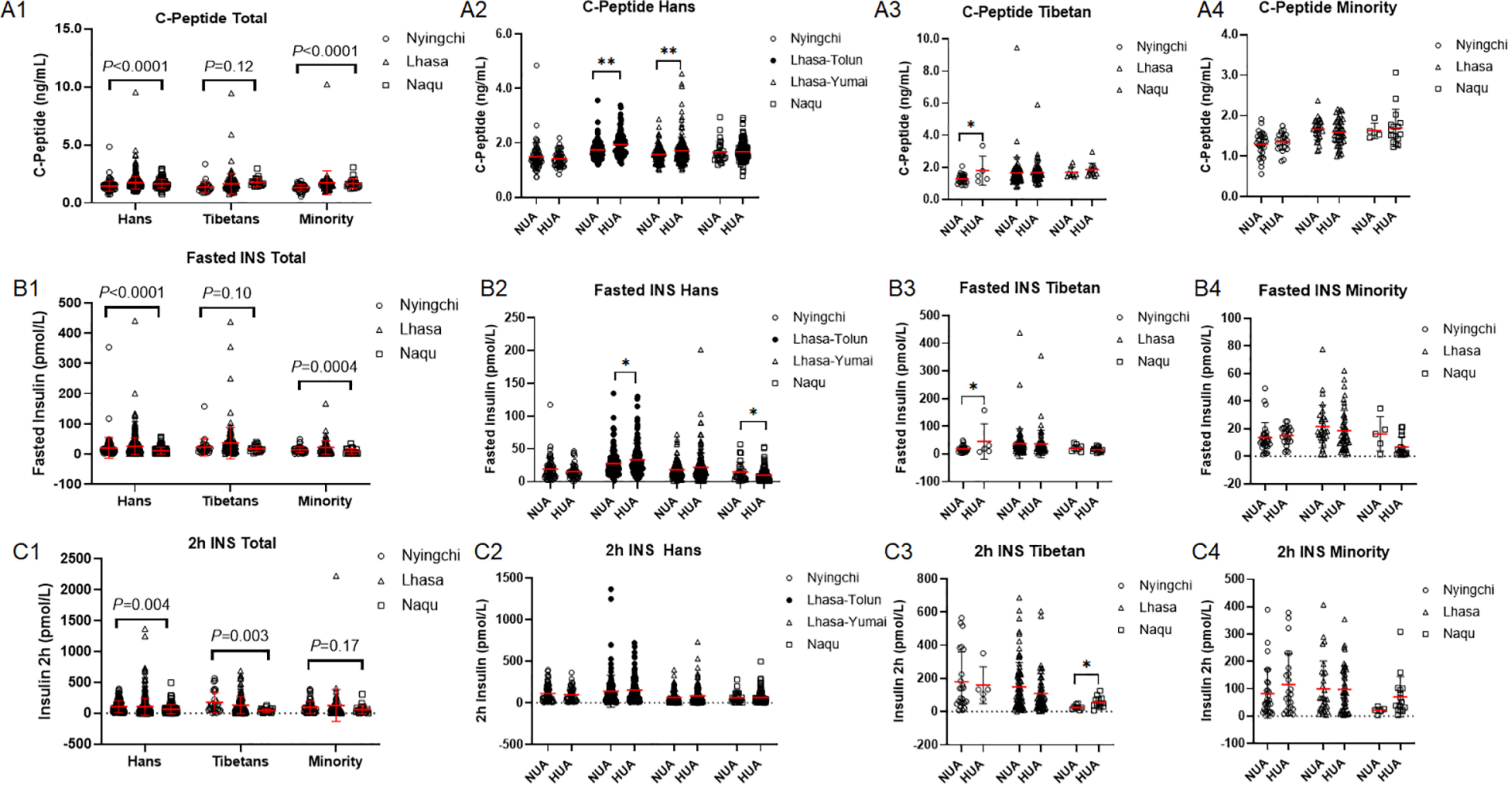
Comparison of insulin secretion capability in oral glucose tolerance test among the Hans, Tibetans, and Minorities in the three altitudes of Tibetan region **(A1–C1)**, and between the respective subpopulations with normal uric acid (NUA) or high uric acid (HUA) levels in Tibet region **(A2–4, B2–4, C2–4)**. *P* values denote the difference among the different counties in the same ethnicity as analyzed by one-way ANOVA. *P < 0.05, **P < 0.01 *vs.* NUA by unpaired t test.

Since the glucose is modulated by insulin, we thus detected insulin secreting ability in β cells in the three cohorts of populations. C-peptide was increased with the altitude exclusively in the Hans and the Minority (both: *P* < 0.0001) rather in the Tibetans (*P* = 0.12) (**Figure 5A1**). Correspondingly, fasted insulin was increased with the altitude exclusively in the Hans (*P* < 0.0001) and the Minority (*P* = 0.0004) rather in the Tibetans (*P* = 0.10) (**Figure 5B1**). By contrast, both the Tibetan and the Hans showed decreased 2h insulin levels with the altitude (Hans: *P* = 0.004; Tibetan: *P* = 0.003) at similar magnitude (*P* = 0.07) (**Figure 5C1**). Surprisingly, the Minority did not show any change of OGTT 2h insulin level with the attitude (*P* = 0.17) (**Figure 5C1**).

Intriguingly, the Hans had higher levels of C-peptide (**Figure 5A2**) and fasted insulin (**Figure 5B2**) in HUA than in NUA group in Lhasa and Naqu, whereases the Tibetans differed in the two parameters between HUA and NUA group only from Nyingchi (**Figures 5A3, B3**). HUA did not differ from NUA in 2h insulin levels in both the Hans and Tibetans from different altitudes, except higher 2h insulin level in HUA than in NUA in Tibetans from Naqu (**Figures 5C2, C3**). Surprisingly, the Minority did not differ between the HUA and NUA in any of the above parameters in any attitude (**Figures 5A4, C4**).

### 3.6 SUA was not associated the decreased IR with the altitude in the 3 ethnic populations

**Figure 6:**
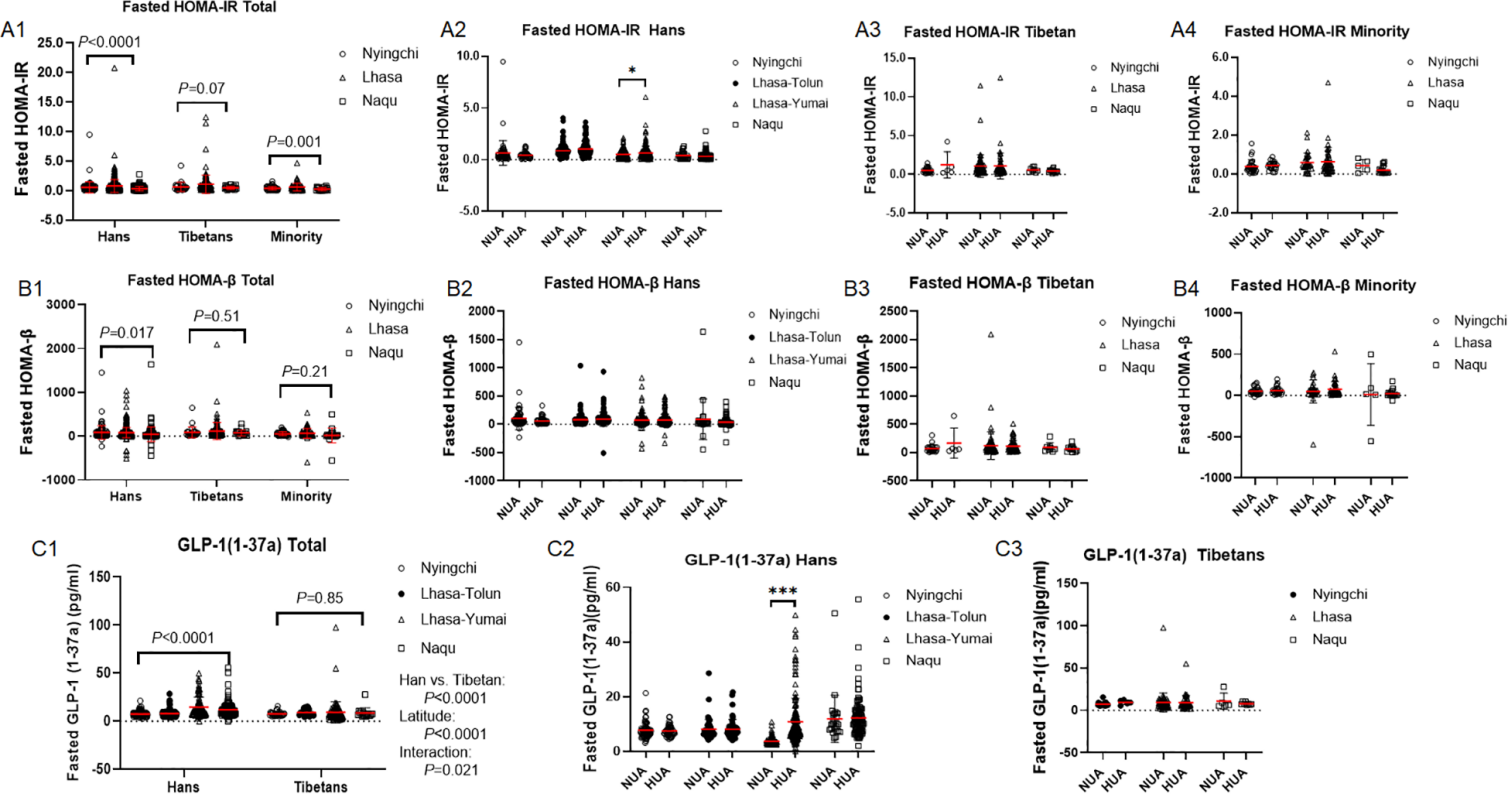
Comparison of fasted insulin resistance (IR) and pancreatic islet secreting capacity among the Hans, Tibetans, and Minorities in three altitudes of Chinese Tibetan region **(A1–C1)**, and between the respective subpopulations with normal uric acid (NUA) or high uric acid (HUA) levels in Tibet **(A2–4, B2–4, C2–3)**. HOMA-IR: Homoeostasis Model Assessment of IR. HOMA-β: Homoeostasis Model Assessment of β-cell function. GLP-1 (1-37a): glucagon-like peptide 1 (1-37) amide. *P* values denote the difference among the different counties in the same ethnicity as analyzed by one-way ANOVA. *P < 0.05, ***P < 0.001 *vs.* NUA by unpaired t test.

Fasted HOMA-IR was declined with the altitude in both the Hans (*P* < 0.0001) and the Minorities (*P* = 0.001) rather in the Tibetans (*P* = 0.07) (**Figure 6A1**). However, 2h HOMA-IR was declined with the altitudes simultaneously in the Hans (*P* = 0.002), the Tibetans (*P* = 0.002) and the Minority (*P* = 0.041) at similar magnitude between the Hans and the Tibetans (*P* = 0.08) (**Supplementary Figure 2B1**). In alignment with higher XOR in HUA than in NUA in the Hans from Lhasa-Yumai (**Figure 2F**), HUA group also had higher fasted (**Figure 6A2**) and 2h HOMA-IRs (**Supplementary Figure 2B2**) than NUA group exclusively in Hans from Lhasa-Yumai, rather than in either the Tibetans (**Figures 6A3, B3**) or the Minority (**Figures 6A4, B4**).

Insulin secretion is modulated by the interaction of pancreas β-cells and α-cells (41, 42), thus we compared fasted and 2h β-cell function as represented by HOMA-β, and fasted α-cell function as represented by GLP-1(1-37a) between the Hans and Tibetans. Fasted HOMA-β was decreased with the altitude exclusively in the Hans (*P* = 0.017) rather in the Tibetans (*P* = 0.51) (**Figure 6A1**). However, 2h HOMA-β did not change with the altitude in both the Hans (*P* = 0.15) and the Tibetans (*P* = 0.72) at similar magnitude (*P* = 0.13) (**Supplementary Figure 2B1**). HUA did not distinguish from NUA in fasted HOMA-β in both the Hans (**Figure 6A2**) and the Tibetans (**Figure 6A3**) in the three altitudes. Nonetheless, lower 2h HOMA-β was also found in HUA than in NUA from the Tibetans in Lhasa (**Supplementary Figure 2B3**).

Importantly, GLP-1(1-37a), a weak insulinotropic hormone (43), was remarkably increased with the altitudes exclusively in the Hans (*P* < 0.0001) rather in the Tibetans (*P* = 0.85), resulting in dramatic difference between the two ethnics (F (1,591)=5.483, *P=*0.020), within the altitudes (F (3,591) = 8.861, *P* < 0.0001), and interactions between the ethnics and the altitudes (F(3,591)=4.636, *P=*0.003) (**Figure 6C1**). Particularly in the Hans from Lhasa-Yumai who stayed the shortest time in Lhasa, GLP-1(1-37a) level was extremely higher in HUA than in NUA group (**Figure 6C2**). Notwithstanding the transient increase, GLP-1(1-37a) did not differ between HUA and NUA groups in either the Hans or the Tibetans from other three counties (**Figures 6C2, C3**). The truncated active GLP-l (7-36) secreted from the intestine had potent ability to stimulate insulin release, however, 2h OGTT GLP-l (7-36) level was not distinguishable from the fasted GLP-l (7-36) in total 3 ethnical populations (**Supplementary Figure 3A**) or in single ethnical population (**Supplementary Figure 3B**) in Nyingchi.

### 3.7 SUA was associated the distinctive hepatic metabolism between the Hans/Minority and the Tibetans

**Figure 7:**
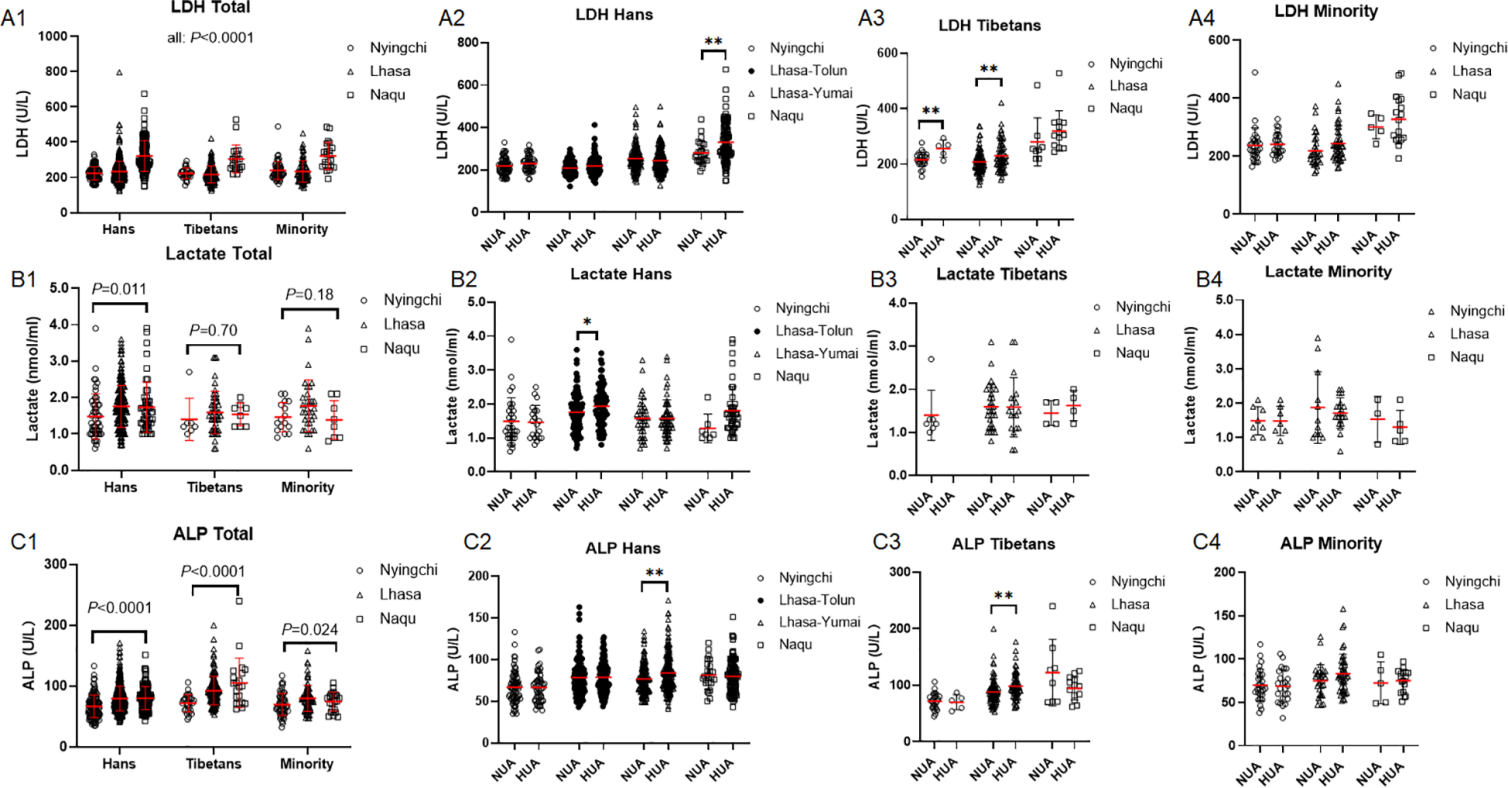
Comparison of hepatic metabolic capacity among the Hans, Tibetans, and Minorities in three altitudes of Chinese Tibetan region **(A1–C1)**, and between the respective subpopulations with normal uric acid (NUA) or high uric acid (HUA) levels in Tibet **(A2–4, B2–4, C2–4)**. LDH: lactate dehydrogenase, ALP: alkaline phosphatase. *P* values denote the difference among the different counties in the same ethnicity as analyzed by one-way ANOVA.*P < 0.05, **P < 0.01 *vs.* NUA by unpaired *t* test.

The change of glucose metabolism may finally affect the overall hepatic metabolism. Thus, we examined hepatic zymogram in the three ethnic cohorts. Lactate dehydrogenase (LDH) levels were increased with the altitudes in the 3 ethnic populations (all: *P* < 0.0001) at similar magnitude (Hans *vs.* Tibetans: *P* = 0.24; Hans *vs.* Minority: *P* = 0.50; Tibetans *vs.* Minority: *P* = 0.07) (**Figure 7A1**). By contrast, lactate was increased with the altitude only in the Hans (*P* = 0.011), but neither in the Tibetans (*P* = 0.70) nor in the Minorities (*P* = 0.18) (**Figure 7B1**). It indicated that the LDH-produced lactate be possibly rapidly mobilized and metabolized in the Tibetans and Minorities.

The HUA group had higher LDH level than NUA group from Nyingchi to Lhasa in the Tibetans (**Figure 7A3**), whereas the difference of LDH level between HUA and NUA only occurred at highest altitude of Naqu in the Hans (**Figure 7A2**). The Minority did not show any difference of LDH between HUA and NUA in any of the three altitudes (**Figure 7A4**). It indicated that he Tibetans acquired the metabolic switch toward glycolysis with elevated LDH level at relative lower altitude, i.e., Nyingchi. On the contrary, HUA group had higher lactate level than NUA group exclusively in the Hans of Lhasa-Tolun (**Figure 7B2**) rather than in the Tibetans (**Figure 7B3**) or the Minority (**Figure 7B4**).

Alkaline phosphatase (ALP) levels were increased with the altitude in the Hans (*P* < 0.0001), Tibetans (*P* < 0.0001), and Minorities (*P* = 0.024) with higher level in the Tibetans than in the Hans and Minorities (both *P* < 0.0001), but comparable levels between the Hans and the Minorities (*P* = 0.93) (**Figure 7C1**). The higher ALP level in HUA *vs.* NUA only occurred in the Hans of Lhasa-Yumai (**Figure 7C2**) and in the Tibetans of Lhasa (**Figure 7C3**).

Arteriosclerosis index (AI) was greatly increased with the altitude especially in the Minority (*P* = 0.001), the Tibetans (*P* = 0.037), but not in the Hans (*P* = 0.11). The Hans (*P* = 0.0005) and the Minority (*P* = 0.008) had higher AI than the Tibetans, without any difference within each other (*P* = 1.00). Except the Hans in Nyingchi, HUA had higher AI than NUA group in both the Hans and the Tibetans from all the 3 altitudes. However, in the Minority population, HUA did not distinguish with NUA group in AI in any altitude (**Supplementary Figure 4A1**). Similar findings in plasma levels of protein and fat metabolic products were available in **Supplementary Figure 5**.

### 3.8 SUA was more associated with reduced FEUA with the altitude in the Hans than in the Tibetans or Minority

**Figure 8:**
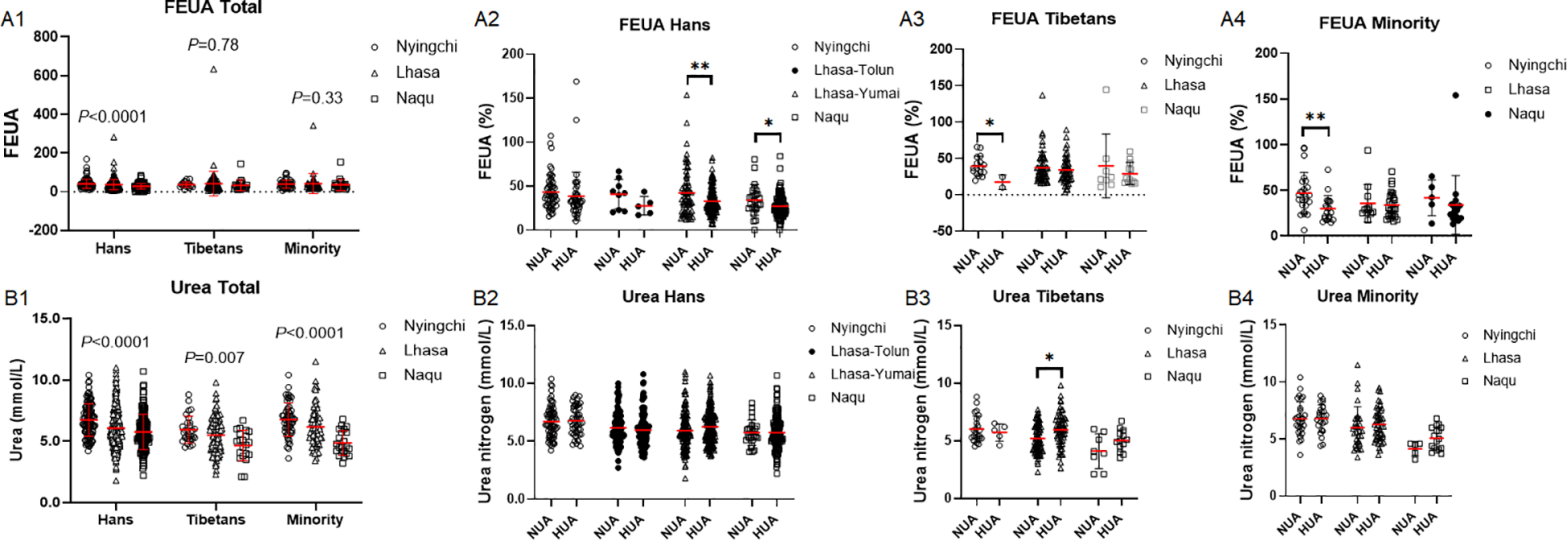
Comparison of renal excreting capacity of uric acid among the Hans, Tibetans, and Minorities in three altitudes of Tibetan region **(A1–B1)**, and between the respective subpopulations with normal uric acid (NUA) and high uric acid (HUA) levels in Tibet **(A2–4, B2–4)**, FEUA: fractional excretion of uric acid. *P* values denote the difference among the different counties in the same ethnicity as analyzed by one-way ANOVA.*P < 0.05, **P < 0.01 *vs.* NUA by unpaired *t* test.

SUA level depends on the UA production and renal filtration rate. FEUA was decreased with the altitude in the Hans (*P* < 0.0001) but neither in the Tibetans (*P* = 0.78) nor in the Minority (*P* = 0.33) (**Figure 8A1**). In the Hans, FEUA was reduced in HUA than in NUA group at higher altitudes of both Lhasa-Yumai and Naqu (**Figure 8A2**). Unexpectedly, in both the Tibetans and the Minorities, lower FEUA was found in HUA vs. NUA group at lower altitude of Nyingchi (**Figures 8A3, 4**).

Although the urea levels were declined with the altitude in the Hans (*P* < 0.0001), the Tibetans (*P* = 0.007) and the Minorities (*P* < 0.0001), the urea level was lowest in the Tibetans (Hans *vs*. Tibetans: *P* < 0.0001; Hans *vs.* Minority: *P* = 0.20; Tibetans *vs.* Minority: *P* = 0.010) (**Figure 8B1**). It did not differ between the NUA and HUA groups in the Hans or the Minority (**Figures 8B2, 4**), however, HUA group showed higher urea level than NUA group in the Tibetans of Lhasa (**Figure 8B3**).

**Figure 9:**
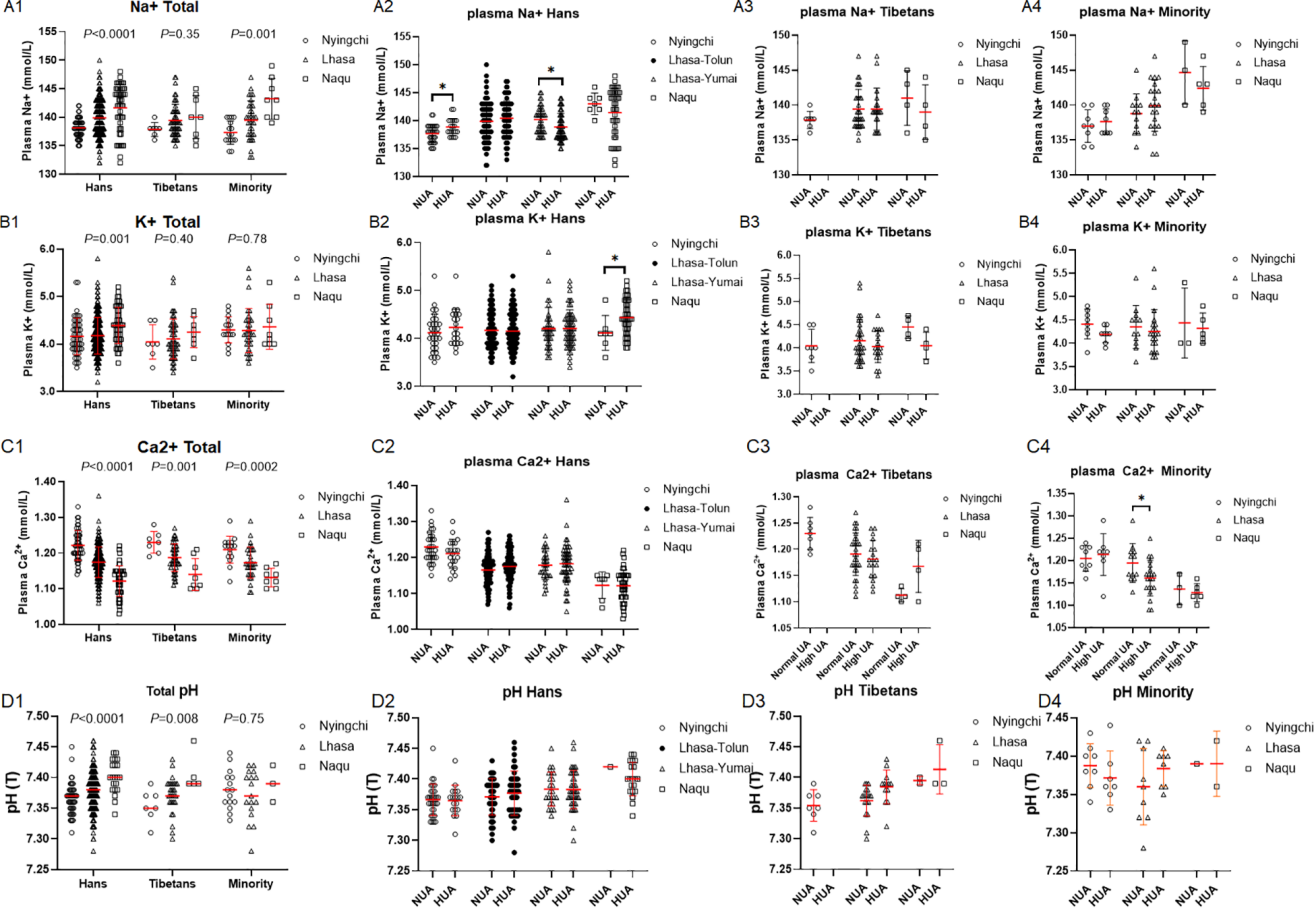
Comparison of plasma electrolytes concentrations among the Hans, Tibetans, and Minorities in three altitudes of Tibetan region **(A1–D1)**, and between the respective subpopulations with normal uric acid (NUA) and high uric acid (HUA) levels in Tibet **(A2–4, B2–4, C2–4, D2–4)**. *P* values denote the difference among the different counties in the same ethnicity as analyzed by one-way ANOVA. *P < 0.05 *vs.* NUA by unpaired *t* test.

Plasma Na^+^ levels were elevated with the altitude in the Hans (*P* < 0.0001), the Minority (*P* = 0.001), but not in the Tibetans (*P* = 0.35) (**Figure 9A1**), while plasma K^+^ level was only elevated with the altitude in the Hans (*P* = 0.001) (**Figure 91**). Furthermore, plasma Ca^2+^ levels were simultaneously decreased with the altitudes in all the three ethnic populations (all *P* < 0.001) (**Figure 9C1**). None of total plasma Na^+^ (F (2, 498) = 1.032, *P* = 0.36), K^+^ (F (2, 100) = 1.945, *P* = 0.15), or Ca^2+^ levels (F (2, 494) = 1.423, *P* = 0.24) of the 3 altitudes differed among the three ethnic populations (**Figures 9A1, B1, C1**). In urine electrolytes, although some variations in either the Na^+^ or K^+^ concentrations among the three ethnics (**Supplementary Figures 6A, B**), urine Ca2+ concentrations in the Hans and the Tibetans rather than in the Minority were invariably decreased with the increase of the altitudes (**Supplementary Figure 6C**).

Because we randomly selected the blood samples to perform the venous gas analysis, the total 7 samples in the Tibetans from Nyingchi happened to be categorized into NUA groups. In spite of the missing gas data in HUA group, a decrease of plasma Na^+^ concentration in Lhasa-Yumai in contrast with an increase of K^+^ concentration in Naqu were found in HUA relative to NUA group exclusively in the Hans (**Figures 9A2, B2**). A decrease of plasma Ca_2_^+^ concentration in HUA relative to NUA group was also found only in the Minority of Lhasa (**Figure 9C4**).

In alignment with the above findings, pH values were elevated with the altitude in the Hans (*P* < 0.0001), the Tibetans (*P* = 0.008), but not in the Minority (*P* = 0.75) (**Figure 9D1**), indicating the occurrence of respiratory alkalosis with the severity of hypoxia (44). It did not differ between HUA and NUA groups from all the 3 ethnic populations in all the 3 altitudes (**Figures 9D2-4**).

### 3.9 SUA was associated with the severity of brain injury depending on the ethnics and exposure time

**Figure 10:**
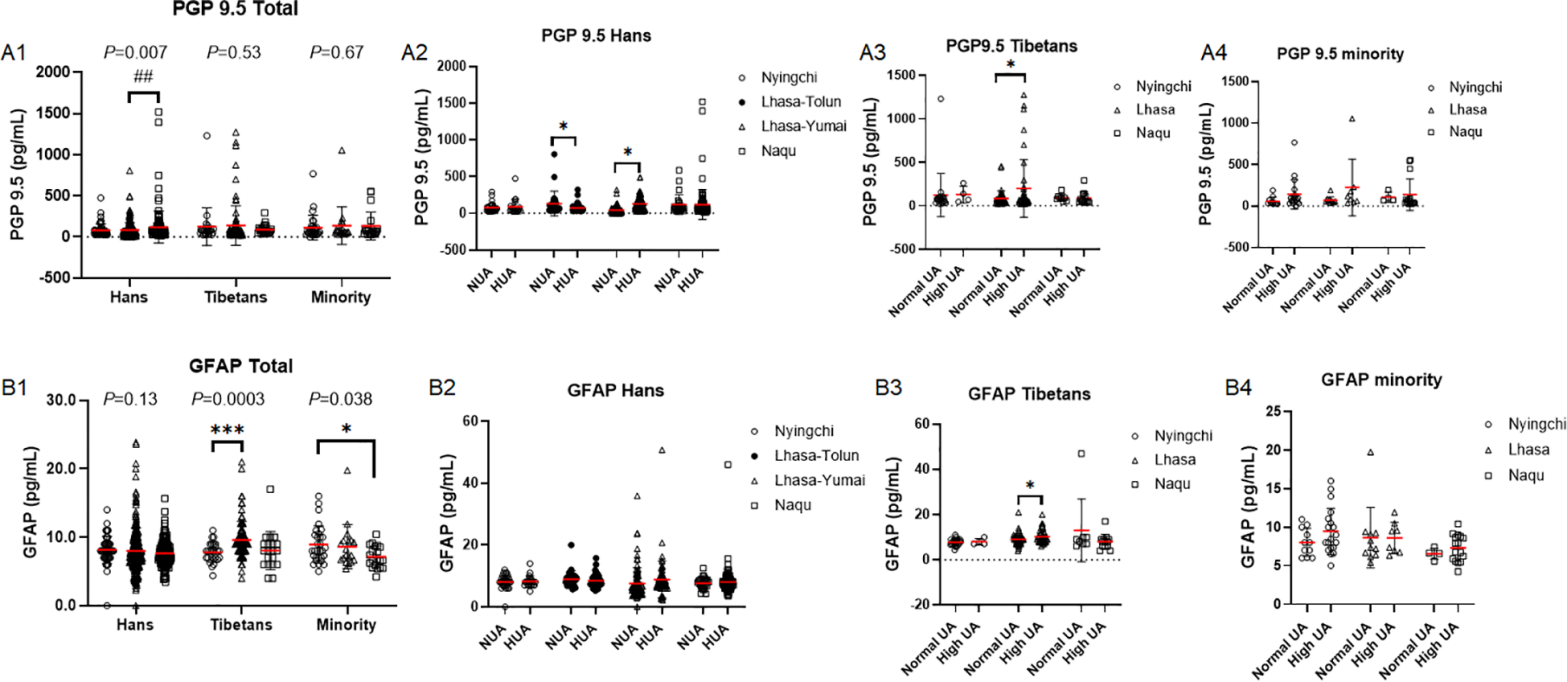
Comparison of brain injury biomarkers among the Hans, Tibetans, and Minorities in the three altitudes of Chinese Tibetan region **(A1–B1)**, and between the respective subpopulations with normal uric acid (NUA) or high UA (HUA) levels in Tibet **(A2–4, B2–4)**, PGP9.5: Protein Gene Product 9.5, GFAP: glial fibrillary acidic protein. *P* values denote the difference among the different counties in the same ethnicity as analyzed by one-way ANOVA. *P < 0.05, ***P < 0.001 *vs.* Nyingchi or NUA; ##*P* < 0.01 *vs.* Lhasa by unpaired *t* test.

The pan-axonal marker PGP 9.5 levels was increased with the altitude in the Hans (*P* = 0.007) with higher level in Naqu than in Lhasa, in contrast, PGP9.5 level did not change with the altitude in the Tibetans (*P* = 0.53) or in the Minority (*P* = 0.68) (**Figure 10A1**). Even so, there was no difference of PGP 9.5 levels among the three ethnic populations in three altitudes (F(2, 613)=1.518, *P* = 0.22) (**Figure 10A1**).

Intriguingly, opposite changes of PGP 9.5 levels in HUA vs. NUA groups between Lhasa-Tolun and Lhasa-Yumai were found in the Hans. That is, lower PGP 9.5 level was found in HUA *vs.* NUA group in Lhasa-Tolun, in contrast higher PGP 9.5 level in HUA *vs.* NUA in Lhasa-Yumai (**Figure 10A2**). Similar to the Hans in Lhasa-Yumai, higher PGP 9.5 level in HUA *vs.* NUA was found in Tibetans from Lhasa (**Figure 10A3**). Surprisingly, in both the Hans and the Tibetans, PGP 9.5 levels did not differ between the NUA and HUA groups in either Nyingchi or Naqu (**Figure 10A2, 3**). Although the enrolled population were relatively small in the Minority, an increased tendency of PGP 9.5 levels in HUA *vs.* NUA in Nyingchi and Lhasa were still revealed (**Figure 10A4**).

Contrary to PGP 9.5, the astrocyte marker GFAP remained unchanged with the altitude in the Hans (*P* = 0.13), however, it was increased significantly with the altitude in the Tibetans (*P* = 0.0003) in contrast with a decrease with the altitude in the Minority (*P* = 0.038) (**Figure 10B1**). There was not significant difference of GFAP levels among the three ethnic populations (Mixed-effects analysis, *P* = 0.22), but significant difference within the altitude (F(2, 413) =4.258, *P* = 0.015), and interactions between the altitudes and ethnics (F(4, 196)=3.122, *P* = 0.016) (**Figure 10B1**). The striking contrast of the changes of GFAP *vs.* PGP 9.5 between the Hans and the Tibetans/Minority suggested that the astrocytes were more liable to be affected by hypoxia in the Tibetans/Minority than in the Hans, by contrast, neural axons were more susceptible to hypoxia in the Hans than in the Tibetans/Minority. Furthermore, the invariably elevations of both GFAP and PGP 9.5 in HUA *vs.* NUA groups in the Tibetans suggested the overall brain injury in their long-term highland adaption even with higher serum UA production. By contrast, in the Minority upon acute highland adaption, GFAP was paradoxically decreased in Naqu as compared with that in Nyingchi (**Figure 10B1**). Considering the Minority had higher serum UA level in Naqu than in Nyingchi (**Figure 2A**), the data was in favor of beneficial effect of serum UA in highland adaption of the Minority.

## 4 Discussion

The prevalence of hyperuricemia in Chinese Tibet region has engendered great concern in public health even though great effort has been paid on the lifestyle intervention (1). In this following study, to our surprise, XOR activity was not in parallel with the increase of SUA in the 3 ethnic populations, strongly suggesting an alternative mechanism underlying the etiology of hyperuricemia at Tibetic plateau. This study points to several potential mechanisms, including higher XOR activity, less FEUA capability, higher ALP activity, and more severe polycythemia, may account for the high prevalence of hyperuricemia in the immigrant Hans. In addition to corroborating our previous biochemistry findings between the Tibetans and the Hans (1), we provided amount evidence that the Minority who immigrated from southwest China resembled the Tibetans more than the Hans in adaptive response to hypoxia.

Our results challenge the conventional paradigm of high prevalence of hyperuricemia association with MS by demonstrating that the Hans had improved IR with the altitude irrespective of the escalation of serum UA levels. Moreover, we first identified that GLP-1 (1-37a) may be involved in adaptive metabolism of glucose with the altitudes exclusively in the Hans. Intriguingly, the diverse manifestation of brain injury biomarkers between the Hans and the Tibetans strongly highlights the beneficial effect of SUA in acute adaptive response of the Hans in contrast with the detrimental effect of SUA in the evolutionary assimilation of the Tibetans in hypoxic Tibet.

### 4.1 Hyperuricemia was not entirely attributed by XOR activity or IR in the three ethnic populations

UA is considered to be an effective antioxidant, so the elevated SUA level encountered in people with MS was considered as a compensatory mechanism counteracting the increased oxidative stress associated with MS (45). In alignment with significantly higher crude prevalence of diabetes in the Hans (14.7%) than in the Chinese Tibetans (4.3%) or Muslims (10.6%) (46), SUA was associated with glucose metabolism exclusively in the Hans instead of in the Tibetans (**Supplementary Table 1**). In several subpopulations of the study, hyperuricemia is not associated with increased XOR activity. It could be explained by that approximately 70% of urate is excreted in the urine, with the remainder occurring via the gastrointestinal tract (47). Therefore, higher XOR activity could only determine more urate production, but could not reflect the overall systemic exchange of urate.

However, in contrast to these data, fasted IR as represented by HOMA-IR was inversely decreased with the altitude in both the Hans and the Minorities, but not in the Tibetans (**Figure 6A1**). The improved IR was further validated by the improved OGTT 2h HOMA-IR with the altitude in the three ethnic populations (**Supplementary Figure A2**). Our findings recapitulated the reports that prolonged residence at high altitudes led to reduced plasma glucose levels mediated by improved insulin sensitivity and augmented peripheral glucose disposal (48). Considering the physical items except the serum UA were within normal physiological ranges in all the 3 ethnic cohorts, the discrepant findings against MS highlight a multifaceted etiology of the hyperuricemia with the ascending altitude.

This hypothesis was strengthened by the increasing plasma GLP-1(1-37a) levels with the altitude exclusively in the Hans rather in the Tibetans. The release of proglucagon-derived peptides from pancreatic α-cells is regulated by autofeedback through glucagon and GLP-1 (43). The above data indicate that proglucagon in the pancreas α cells undergo increased processing with the altitude only in the Hans. Since GLP-1(1-37a) is truncated from the same major proglucagon fragment as the glucagon in the α cells (41, 49, 50), it is tempting to speculate a concurrent increase of glucagon levels with the altitudes and possible higher glucagon level in HUA than in NUA group from the Hans of Lhasa-Yumai. These findings were in accordance with higher fasted and 2h glucose levels, as well as higher HbAC1 level in HUA than in NUA group from the Hans in Lhasa-Yumai (**Figures 4A2, B2, C2**). Even in the Hans, SUA was more associated with pancreas α cell function as represented by higher GLP-1(1-37a) in HUA *vs.* NUA in Lhasa-Yumai (**Figure 6C2**) than with β cell function as represented by comparable fasted HOMA-β level between HUA and NUA groups (**Figure 6B2**). Our study revealed an unexplored α cells function in the acute adaptive change of glucose metabolism in the Han immigrants.

### 4.2 Hyperuricemia may be associated with heightened PPP in both the RBCs and chondrocytes in all the three ethnic populations

The PPP not only contributes to cell proliferation through the production of ribose-5-phosphate, but also to the biosynthesis and redox homeostasis through the production of the largest source of nicotinamide adenine dinucleotide phosphate (NADPH) (51). The purinergic system also participates in driving RBCs metabolic adaptations to high altitude hypoxia (52) and provides protection from oxidative stress (53). The incidence of hyperuricemia was significantly higher in Tibetan adults with HAPC than without HAPC (2). Circadian rhythms of glycolysis and PPP metabolites in RBCs was required to prevent cellular damage caused by the daily auto-oxidation of HGB by maintaining the redox rhythmicity in RBCs (54). In our study, both the HGB and RBC levels were dramatically elevated in all the three ethnic populations with the altitudes, reaching the threshold value of polycythemia in Naqu (**Figure 3B1**). It was possible that erythrocyte-derived UA may confer significant protection of RBCs against oxidative stress in high altitudes of Tibet. Since the extra and intracellular UA could be actively transported through the human erythrocyte membrane (55), the rapid increase of SUA with the altitude may be originated from the increased numbers of RBCs against the hypoxia (52, 56, 57), by which HGB was also a risk factor for hyperuricemia (3).

In accordance with the metabolic centrality of PPP in RBCs, glucose metabolism by the PPP is necessary to maintain chondrocytes viability and endochondral ossification in an avascular hypoxic milieu of the growth plate of long bones. By promoting glutathione recycling, PPP-derived NADPH scavenges reactive oxygen species (ROS) generated in oxidative stress, resulting in proteostasis and thereby the chondrocytes survival (58). Amazingly, extra-erythrocyte chondrocytes were capable to produce massive amounts of HGB in responding to hypoxia with markedly left-shifted p50 for short-range supply (59). It is tempting to speculate that the ectopic expression of HGB in the chondrocytes also underwent metabolic PPP switch to remain its survival under the extreme severe hypoxia environment of Tibet. Our hypothesis was substantiated by the consensus that the intra-articular gout stone be usually found around the femoral condyle and joint space where the chondrocytes exist (60).

### 4.3 Hyperuricemia may be associated with an increased bone turnover in all the three ethnic populations

The accelerated decrease of plasma Ca^2+^ level with the ascending altitude were apprehensive (**Figure 9C**). Firstly, acute hypoxia induces compensatory tachycardia with excessive CO_2_ excretion, which in turn lead to respiratory alkalosis as represented with higher blood pH (**Figure 9D**). Secondly, the elevated levels of plasma proteins, such as the increased levels of prealbumin and albumin with the altitude (**Supplementary Figures 5A, B**) carry an increased negative charge, which bind more free calcium leading to the decrease of ionic calcium. Thirdly, the raised ALP activity with the altitudes, predominant in the Tibetans (**Figure 7C**), could represent the increased bone turnover against hypoxia (61) that may increase the risk of osteoporosis (62) to as high as 28.3% in Tibet (62). ALP was involved in the regulation of purinergic signaling by participating in the degradation of extracellular nucleotides (63), therefore, it may promote SUA production through PPP activation (45).The two biological effects of ALP may account for the risk of SUA/creatinine ratio for osteoporosis (64). By contrast, inhibition of XOR activity in rat osteoblast cells promoted osteoblast differentiation and led to increased bone formation (65). Thus, the lower plasma Ca2+ levels in concurrent with higher SUA levels with the altitudes indicated the progressive worsening of osteoporosis as a result of augmented bone turnover in Tibet.

### 4.4 Hyperuricemia was more associated with the renal electrocytes exchange ability in the Hans than in the Tibetans

Several studies revealed that IR is inversely related to 24 h urinary clearance, therefore, one mechanism linking hyperinsulinemia (a consequence of IR) with hyperuricemia is the reduced renal excretion of UA (66). In our study, FEUA level was decreased with the altitude exclusively in the Hans, but neither in the Tibetans nor in the Minority (**Figure 8A1**). In the Hans from moderate altitude of Lhasa to extreme high altitude of Naqu, HUA group had reduced FEUA than NUA group while comparable urea levels as the NUA group (**Figure 8A2**). By contrast, in the Tibetans, FEUA remained unchanged between the HUA and NUA groups in both Lhasa and Naqu, but was reduced in HUA *vs.* NUA only in lower altitude of Nyingchi (**Figure 8A3**). These data suggested that hyperuricemia was exclusively associated with renal UA filtration function in the Hans rather than in the Tibetans or the Minority.

In accordance, plasma Na^+^ levels were increased with the ascending altitude in both the Hans and the Minority, but not in the Tibetans, while plasma K^+^ level was only increased with the ascending altitude in the Hans (**Figures 9A1, B1**). Although the urine electrocytes did differ among the three different altitudes occasionally in the some of the three ethnic populations, they did not change significantly in line with the ascending altitude (**Supplementary Figure 6**), it seemed that renal electrocytes exchanges were more severely impaired in the Hans than in the Tibetans with the ascending altitude, which may account largely for the increased hyperuricemia with the altitude in the Hans.

### 4.5 Hyperuricemia may be associated with brain injury in both the Hans and the Tibetans

PPP is a glucose shunt pathway that generates the reducing agent NADPH and provides precursors for nucleotide synthesis (67). Neurons are more susceptible to oxidative stress (68, 69). Thus, the PPP represents a major defense mechanism in neurons that protects them against oxidative stress. Within a few months after the inland Hans ascended to Lhasa-Yumai, the exposure to acute hypobaric hypoxia could cause severe oxidative brain injury. As a compensate, XOR was immediately activated in the Hans to rapidly produce large amount of UA to ameliorate the cerebral oxidative stress in Lhasa-Yumai (**Figure 2F**). Even so, the brain injury could not be completely reversed, so the markers of brain injury in HUA group were still higher than those in NUA group (**Figure 10A2**). When the Hans gradually adapted to the cruel environment as seen in Lhasa-Tolun after several years’ stay, rather than through the modulation of XOR activity (**Figure 2F**), they raised the level of SUA possibly through increased nucleoside turnover associated with increased erythropoiesis (**Figure 3C2**), the activation of hypoxanthine salvage pathway, or simply facilitated release of urate into the plasma, and most likely through remarkably reduced urate excretion in the urine (**Figure 8A2**). Therefore, when the Hans was assimilated in Tibet for a few years, brain injury was attenuated in HUA than in NUA group (**Figure 10A2**).

With respect to the Tibetans, although XOR level was exclusively increased with the altitudes in the Hans (*P* < 0.0001) instead in the Tibetans (*P* = 0.93), XOR level was significantly higher than that in the Hans from the same altitude of Lhasa. Although XOR levels in the Tibetans was only higher in HUA than in NUA group in Lhasa (**Figure 2G**), both of the two biomarkers of brain injury were higher in HUA than in NUA group in Lhasa in contrast with decreased tendency in Naqu (**Figures 10A3, B3**). It suggested that even though the Tibetans may evolve to obtain better anti-oxidant ability, it was not sufficient to counteract oxidative brain injury in Tibet. Another possibility was that XOR activity, rather than the hyperuricemia itself, was associated with prooxidant injury especially within the vascular bed (70). These findings were recapitulated in our study. Firstly, in the Tibetans with the ascending altitudes, the change of GFAP (**Figure 10B1**) was in alignment with that of XOR (**Figure 2E**), but quite different from the invariably increased serum UA level (**Figure 2A**), indicating that the cerebral prooxidant processes was in accordance with the XOR activity rather than with the hyperuricemia. Secondly, with the ascending altitudes, AI remained unchanged in the Hans, but was slightly elevated in the Tibetans (**Supplementary Figure 4A1**), coincident with the moderate changes of XOR rather than with the worsened hyperuricemia, reinforcing the prooxidant injury of XOR activity rather the hyperuricemia on vascular cells.

This study has some limitations. First, we did not examine all the indicated parameters in all the enrolled population to give a complete picture of the metabolic changes, especially in the Minority. Secondly, we did not clarify if these alterations in SUA represent a consequence of brain injury or causatively contributes to it in high altitude adaption in an animal model. Furthermore, longitudinal study should be carried out to determine whether these adaptive responses could resolve and how long it requires to fully revert to normal level after relocating to low altitude. Therefore, cautious would be taken in the interpretation of all the results.

Overall, this study points to a beneficial effect of hyperuricemia on brain injury in high-altitude adaption rather than the risk factor for MS in the inland immigrants, challenging the traditional strategy aiming at marker correction in certain situation which could be translated into critically ill patients under pathological hypoxia. Moreover, the Tibetans was entitled with distinguished biochemical characteristics and outcomes in comparison with the Hans, dictating distinctive strategy targeting hyperuricemia between the Hans and the Tibetans.

## Data Availability

The raw data supporting the conclusions of this article will be made available by the authors, without undue reservation.
