# Supplementary material for: Hyperuricemia was associated with metabolic response against brain injury instead of metabolism syndrome in Tibet: a cross-sectional analysis
Source: Front Endocrinol (Lausanne). 2026 Apr 23;17:1776220. doi: 10.3389/fendo.2026.1776220 (PMC13149101; doi:10.3389/fendo.2026.1776220)
Supplement: Supplementary file 1 [file DataSheet1.docx]

1. **Supplementary methods**

Arterial Stiffness Index (AI), an indicator of endothelial function, was calculated as AI= [TCHOL-HDL-CH]/HDL-CH by standard calorimetric assays. TCHOL  represents total cholesterol, while HDL-CH is high-density lipoprotein- cholesterol.

1. **Supplementary Result**
   1. **Hyperuricemia was not associated with the** **changes of the peripheral mononuclear blood cells (****P****MBCs)**

To exclude the systemic inflammatory response on brain injury, we also examined compared the numbers of PMBCs by routine blood test. Coincident with heightened serum GFAP, a biomarker of neuroinflammation, the significant increases of monocytes and lymphocytes with the altitude were exclusively observed in the Tibetans (**Fig.S1C1, D1**). All the parameters did not differ between HUA and NUA in any ethnic population from any altitude (**Fig.S1**).

**Suppl.Fig.1** Comparison of peripheral mononuclear blood cells among the three ethnic populations with the ascending altitudes (A1, B1,C1,D1) in Tibet, and between the populations with normal uric acid (NUA) and high uric acid (HUA) levels from respective ethnic populations (A2-D2, A2-D3, A2-D4) in Tibet. *P* values denote the difference among the 3 counties from the same ethnic population as analyzed by one-way ANOVA. ***P*<0.01 *vs.* NUA by unpaired *t* test.

- 1. **Improved IR in contrast with decreased β cells function with the altitude in the Hans rather than in the Tibeta****n**

**Suppl.Fig.2** Comparison of insulin resistance (IR) index (A) and fasted pancreatic islet secreting capacity (B) at 2h oral glucose tolerance test (OGTT) among the three ethnic populations with the ascending altitudes (A1, B1) in Tibet, and between the populations with normal uric acid (NUA) or high uric acid (HUA) levels from the three ethnics (A2-B2, A3-B3, A4-B4) in Tibet. HOMA-IR: Homoeostasis Model Assessment of IR, HOMA-β: Homoeostasis Model Assessment of β-cell function. *P* values denote the difference among the different counties in the same ethnicity as analyzed by one-way ANOVA. **P*<0.05, ****P*<0.001 *vs.* NUA by unpaired *t* test.

The truncated active GLP-l (7-36) secreted from the intestine had potent ability to stimulate insulin release at concentrations of 50-100 pmol/L [[49](#_ENREF_49)]. In our detection, GLP-l (7-36) level from the 3 ethnic populations in Nyingchi was much higher than previously reported in humans that usually below 10 pmol/L with other commercial kits (Cat. No.: EZGLPHS-35K, Millipore)[[50](#_ENREF_50), [51](#_ENREF_51)]. Despite of the incompatibility, the 2h OGTT GLP-l (7-36) was not distinguishable from the fasted GLP-l (7-36) in total 3 ethnical populations (**Suppl.Fig.3A**) or in single ethnical population (**Suppl.Fig.3B**) in Nyingchi. The Tibetans seemed to have lower fasted GLP-l (7-36) level than the other two ethnic populations, but for a few fasted samples (n=3), it did not reach its significance. The detection of plasma GLP-l (7-36) at early time points during OGTT may be more appropriate for the comparison.

**Suppl.Fig.3** Plasma active GLP-1 (7-36) levels were not significantly elevated 2h after oral glucose tolerance test (OGTT) in total 3 ethnical population (A) or in respective ethnicity (B) in Nyingchi.

- 1. **Hepatic metabolism revealed distinctive profile between the Hans/Minority and the Tibetans**

  None of the hepatic γ-glutamyl transferase (GGT) level was changed with the altitude in every ethnic population (Hans: *P*=0.53, Tibetans: *P*=0.58, Minority: *P*=0.40) (**Suppl.Fig.4A1**). HUA had higher AI than NUA group in both the Hans and Tibetans in every altitude (**Suppl.Fig.4A2,3**), but not in the Minority (**Suppl.Fig.4A4**). Total GGT levels in the three altitudes did not differ significantly among the three ethnic populations (F (2, 1096)=1.617, *P* = 0.20) (**Suppl.Fig.4B1**). HUA had higher GGT level than the NUA group exclusively in the Hans from Lhasa-Tolun (**Suppl.Fig.4B2**). It indicated that the GGT may be involved in the etiology of hyperuricemia in the middle altitude of Hans in Lhasa-Tolun after long-term dwelling.

**Suppl.Fig.4** Comparison of arteriosclerosis index (AI) (A) and hepatic γ-glutamyl transferase (GGT) activity (B) among the three ethnic populations with the ascending altitudes (A1, B1) in Tibet, and between the populations with normal uric acid (NUA) or high UA (HUA) levels from the three ethnics (A2-B2, A3-B3, A4-B4) in Tibet. *P* values denote the difference among the 3 altitudes within each ethnicity as analyzed by one-way ANOVA. **P*<0.05 *vs.* NUA by unpaired *t* test.

Prealbumin levels were changed with the altitude in the Hans (*P*<0.0001), the Minority (*P*<0.0001) but not in the Tibetans (*P*=0.29) (**Suppl.Fig.5A1**). Total prealbumin levels in the three altitudes differed significantly among the three ethnic populations (F (2, 1096) = 7.285, *P*=0.001), but comparable between the Hans and the Minority (*P*=0.94) (**Suppl.Fig.5A1**).

Similarly, albumin levels were changed with the altitude in the Hans (*P*<0.0001), the Minority (*P*<0.0001) but not in the Tibetans (*P*=0.39) (**Suppl.Fig.5B1**). Total albumin levels in the three altitudes differed significantly among the three ethnic populations (F (2, 336) = 6.081, *P*=0.003), but comparable between the Hans and the Minority (*P*=1.00) (**Suppl.Fig.5B1**). The data suggested that the protein turnover with the ascending altitude was similar between the Hans and the Minority, but was higher than that in the Tibetans. Nonetheless, HUA had, or intended to have higher prealbumin and albumin levels than the NUA group in both the Hans and the Tibetans in the three altitudes (**Suppl.Fig.5A2-3, B2-3**), while had higher albumin level only in the Minorities from Nyingchi (**Suppl.Fig.5B4**). These findings indicated that magnified protein metabolism be involved in the etiology of hyperuricemia in both the Hans and the Tibetans.

TCHOL level was decreased with the altitude in the Hans (*P*=0.007), but neither in the Tibetans (*P*=0.35) nor in the Minority (*P*=0.21) (**Suppl.Fig.5C1**). TCHO levels in the three altitudes differed significantly among the three ethnic populations (F (2, 1096) = 13.24, *P*<0.0001), highest in the Hans (Hans vs. Tibetans: *P*=0.001; Hans vs. Minority: *P*=0.008) (**Suppl.Fig.5C1**).

By contrast, triglyceride (TG) levels were universally increased with the altitude in the Hans, the Minority, and the Tibetans (all: *P*=0.001), with significant difference among the three ethnicities (F (2, 336) = 7.629, *P*=0.001) (**Suppl.Fig.5D1**). TG levels in the three altitudes were significantly higher in the Hans than in the Tibetans (*P*=0.0004), but comparable between the Hans and Minority (*P*=0.48), the Tibetans and Minority (*P*= 0.07) (**Suppl.Fig.5D1**).

HUA had higher TCHO and TG levels than the NUA group in both the Hans (**Suppl.Fig.5C2, D2**) and the Tibetans (**Suppl.Fig.5 C3,D3**) rather in the Minority (**Suppl.Fig.5C4,D4**) in the three altitudes, indicating that augmented fat metabolism involved in the etiology of hyperuricemia in the Hans and the Tibetans instead of the Minority.

**Suppl.Fig.5** Plasma levels of protein and fat metabolic products among the three ethnic populations with the ascending altitudes (A1, B1,C1,D1) in Tibet, and between the populations with normal uric acid (NUA) or high UA (HUA) levels from the three ethnic populations (A2-D2, A2-D3, A2-D4) in Tibet. *P* values denote the difference among the different counties in the same ethnicity as analyzed by one-way ANOVA. **P*<0.05, ***P*<0.01, ****P*<0.001 *vs.* NUA by unpaired *t* test.

- 1. **Serum UA was not consistently associated with the concentrations of urine electrolytes with the altitude in the 3 ethnic populations**

**Suppl. Fig.6** Comparison of urine electrolytes concentrations among the three ethnic populations with the ascending altitudes in Chinese Tibetan region. *P* values denote the difference among the different counties in the same ethnicity as analyzed by one-way ANOVA.

- 1. **Distinctive** **associations of serum UA with both glucose metabolism and brain injury between** **the Hans and the Tibetans**

Multivariate line regression analysis of the association of glucose or UA metabolism, urine UA and brain injury markers with serum UA revealed absolutely different manifestation between the Hans and the Tibetans in three altitudes (**Suppl.Table**). In the Hans, the biomarkers of glucose metabolism and urine UA were associated with serum UA level, e.g., HbA1C in Nyingchi, fasted glucose and urine UA in Lhasa, XOR and fasted glucose in Naqu. On the contrary, all these biomarkers were absolutely not associated with serum UA in the Tibetans. These corroborative evidences strongly suggested the distinguished etiology of hyperuricemia in the Tibetans from that in the Hans (**Suppl. Table**).

**Suppl. Table** Multivariate line regression analysis of the association of metabolic parameters with serum uric acid in male populations of the Hans and Tibetans in three high-altitude regions of Tibet, China.

| Ethnicity | Location |  | Nyingchi |  |  |  | Lhasa |  |  |  | Naqu |  |
| --- | --- | --- | --- | --- | --- | --- | --- | --- | --- | --- | --- | --- |
|  | Variable | OR | 95% CI (asymptotic) | *P* value |  | OR | 95% CI (asymptotic) | *P* value |  | OR | 95% CI (asymptotic) | *P* value |
|  | GLP-1 | 0.11 | -7.693 to 6.871 | 0.91 |  | 0.31 | -2.817 to 2.049 | 0.75 |  | 1.15 | -1.002 to 3.785 | 0.25 |
|  | XOR | 0.14 | -0.2404 to 0.2090 | 0.89 |  | 0.64 | -0.1559 to 0.07975 | 0.52 |  | 2.32 | 0.03070 to 0.3894 | 0.022* |
|  | fasted glucose | 0.65 | -30.31 to 59.31 | 0.52 |  | 3.09 | 18.19 to 84.14 | 0.003* |  | 2.30 | 2.825 to 37.54 | 0.023* |
|  | OGTT2h glucose | 1.37 | -42.35 to 8.050 | 0.18 |  | 0.06 | -20.01 to 21.33 | 0.95 |  | 0.33 | -13.00 to 18.27 | 0.74 |
| Hans | HbA1C | 2.29 | 10.97 to 173.3 | 0.027* |  | 0.16 | -26.08 to 30.53 | 0.88 |  | 0.36 | -30.76 to 44.51 | 0.72 |
|  | Urine UA | 0.10 | -0.02434 to 0.02209 | 0.92 |  | 3.71 | -0.05906 to -0.01780 | 0.0004* |  | 0.48 | -0.02645 to 0.01616 | 0.63 |
|  | PGP 9.5 | 0.45 | -0.1801 to 0.2836 | 0.65 |  | 0.37 | -0.04974 to 0.07236 | 0.71 |  | 0.01 | -0.07973 to 0.08085 | 0.99 |
|  | GFAP | 0.09 | -10.49 to 11.42 | 0.93 |  | 1.84 | -6.322 to 0.2450 | 0.07 |  | 0.61 | -2.846 to 5.389 | 0.54 |
|  | GLP-1 | 1.32 | -4.347 to 18.79 | 0.21 |  | 0.63 | -2.178 to 1.141 | 0.53 |  | 2.32 | -18.00 to 1.617 | 0.08 |
|  | XOR | 0.62 | -0.2727 to 0.5002 | 0.54 |  | 0.43 | -0.09001 to 0.1391 | 0.67 |  | 0.04 | -0.5388 to 0.5559 | 0.97 |
|  | fasted glucose | 1.06 | -22.13 to 66.75 | 0.30 |  | 0.41 | -36.69 to 55.41 | 0.69 |  | 1.53 | -157.6 to 45.57 | 0.20 |
|  | OGTT2h glucose | 0.47 | -22.85 to 14.58 | 0.65 |  | 1.20 | -39.10 to 9.778 | 0.23 |  | 1.97 | -31.85 to 188.2 | 0.12 |
| Tibetans | HbA1C | 0.95 | -118.3 to 44.91 | 0.36 |  | 0.24 | -55.91 to 71.10 | 0.81 |  | 0.40 | -121.7 to 163.1 | 0.71 |
|  | Urine UA | 1.04 | -0.03213 to 0.01092 | 0.31 |  | 0.71 | -0.01464 to 0.03071 | 0.48 |  | 1.33 | -0.03296 to 0.09330 | 0.26 |
|  | PGP 9.5 | 0.28 | -0.08818 to 0.06761 | 0.78 |  | 1.69 | -0.01212 to 0.1429 | 0.10 |  | 1.82 | -1.865 to 0.3892 | 0.14 |
|  | GFAP | 0.25 | -13.05 to 16.60 | 0.80 |  | 0.77 | -4.685 to 10.58 | 0.44 |  | 1.44 | -11.85 to 37.39 | 0.22 |

Footnote: **P*<0.05, ***P*<0.01, ****P*<0.001. glucagon-like peptide 1 (1-37) amide (GLP-1), xanthine oxidoreductase (XOR), oral glucose tolerance test (OGTT), Glycosylated Hemoglobin, Type A1C (HbA1C), uric acid (UA), Protein Gene Product 9.5 (PGP9.5), glial fibrillary acidic protein (GFAP), γ-glutamyl transferase (GGT), triglyceride (TG), total cholesterol (TCHOL), high-density lipoprotein cholesterol (HDL-CH), low-density lipoprotein- cholesterol (LDL-CH)
